# Scaffold Fabrication Techniques of Biomaterials for Bone Tissue Engineering: A Critical Review

**DOI:** 10.3390/bioengineering9120728

**Published:** 2022-11-24

**Authors:** Sakchi Bhushan, Sandhya Singh, Tushar Kanti Maiti, Chhavi Sharma, Dharm Dutt, Shubham Sharma, Changhe Li, Elsayed Mohamed Tag Eldin

**Affiliations:** 1 Department of Paper Technology, IIT Roorkee, Saharanpur 247001, India; 2 Department of Polymer and Process Engineering, IIT Roorkee, Saharanpur 247001, India; 3 Mechanical Engineering Department, University Center for Research & Development, Chandigarh University, Mohali 140413, India; 4 School of Mechanical and Automotive Engineering, Qingdao University of Technology, Qingdao 266520, China; 5 Faculty of Engineering and Technology, Future University in Egypt, New Cairo 11835, Egypt

**Keywords:** bone tissue engineering, fabrication, biocompatibility, electrohydrodynamic behavior, additive manufacturing techniques, 4D printing, clinical trials

## Abstract

Bone tissue engineering (BTE) is a promising alternative to repair bone defects using biomaterial scaffolds, cells, and growth factors to attain satisfactory outcomes. This review targets the fabrication of bone scaffolds, such as the conventional and electrohydrodynamic techniques, for the treatment of bone defects as an alternative to autograft, allograft, and xenograft sources. Additionally, the modern approaches to fabricating bone constructs by additive manufacturing, injection molding, microsphere-based sintering, and 4D printing techniques, providing a favorable environment for bone regeneration, function, and viability, are thoroughly discussed. The polymers used, fabrication methods, advantages, and limitations in bone tissue engineering application are also emphasized. This review also provides a future outlook regarding the potential of BTE as well as its possibilities in clinical trials.

## 1. Introduction

**Figure 1:**
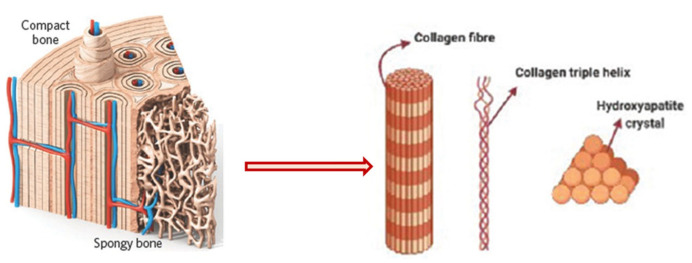
Schematic diagram of bone hierarchical structure. Reprinted with permission from Ref. [8] Copyright 2016, John Wiley & Sons, Ltd.

Human bone is a biocomposite, mainly consisting of inorganic minerals and organic collagen [1]. The hierarchical arrangement of bone contains collagen fibrils with the deposition of hydroxyapatite (Ca_10_(PO_4_)_6_(OH)_2_) nanocrystals [2]. The inner architecture includes cancellous or spongy bone (~80–90%), which is highly vascularized and possesses an interconnected porous structure, whereas the outer architecture is hard compact bone (~10%) due to the high mineral content (Figure 1) [3].

In addition to that, various functions in bone such as bone formation and resorption, mineral homeostasis, and bone repair are performed by bone cells such as osteoblasts, osteocytes, and osteoclasts [4]. Osteoblasts are present on the lining surface of the bone. Their function is to synthesize and secrete the organic matrix of bone, termed osteoid [5]. It is rich in ribosomes, Golgi apparatuses, endoplasmic reticulums, and mitochondria. Active osteoblasts are enclosed in the matrix to form osteocytes, which contain few endoplasmic reticulums and various cytoplasmic organelles [4]. This association between osteocytes and osteoblasts helps in the regulation of mineral ions between the bone matrix and the extracellular space of the bone.

Osteoclasts are scarcely found in normal bone, as they perform the bone resorption process. They contain a few ribosomes, endoplasmic reticulums, mitochondria, Golgi apparatuses, and Golgi vesicles. They release acid phosphatase and collagenase that break down minerals and clear the organic matrix for up to 1–2 µm [6]. The degraded components are absorbed by endocytosis, transported, and extruded into the extracellular space [7].

Bone bears a high load and deformation due to its stupendous mechanical strength and has a significant role in nutrient homeostasis and the regulation of mineral content. Bone disorders due to fracture, calcium deficiency, tumors, or endocrine diseases (e.g., osteoporosis and diabetes) have increased drastically worldwide, making the complete recovery of a patient’s bone a challenging task. There are various stages that occur during the bone healing process [9]. The initial phase is the resting phase, in which cells rest at the fractured site of the bone. Later, the resorption process starts with the accomplishment of osteoclast cells. The bone remodeling process involves the replacement of weak bones with new different bones, resulting in the initiation of the healing process. The formation of bone and its breakdown depend on the coordination of osteoblast and osteoclast cells, respectively. An imbalance between the formation and breakdown of bone cells results in osteoporosis. 

Bone graft surgeries are conducted in 2 million reported cases every year, making it the second most transplanted tissue after blood transfusions [10]. As a result, a tailored alternative is crucially needed to repair bone defects. Autograft sources are considered the gold standard, as they provide osteoinductivity, osteoconductivity, osteogenesis, and osteointegration to the bones, but donor site morbidity and scar formation due to multiple surgeries limit their application. Moreover, allograft and xenograft sources are readily available, but there is a chance of disease transmission. To eliminate this, tissue engineering is a multidisciplinary field that helps in the formation of a construct incorporating a polymer-based scaffold, osteogenic cells, and growth factors [11]. In this context, BTE offers the synergistic effects of cells, growth factors, and biomaterials to the construct for the repair of damaged or diseased bone tissue. An ideal bone scaffold should be biomimetic and biodegradable, have porous support cell attachment and proliferation as well as differentiation, and possess adequate mechanical strength to persist at the implantation site, minimizing the risks of immunogenicity [12]. 

There are various fabrication techniques that have been developed to improve osteogenesis. The pore architecture and porosity have a substantial impact on the mechanical and biological properties of bone tissue. Therefore, the choice of a fabrication method should be able to create repeatable scaffolds with precise hierarchical porous structures [13]. Moreover, the infusion of heat-labile drugs, growth factors, and biological components in the scaffolds to improve bone healing is of the utmost importance. To accomplish this, advanced techniques are used for the fabrication of scaffolds that meet the desired criteria. This review focuses on the fabrication techniques designed to produce bone scaffolds supporting osteogenic behavior. These techniques include the conventional method, the electrohydrodynamic and additive manufacturing techniques, and various advanced methods of scaffold fabrication, which are discussed.

## 2. Conventional Methods

### 2.1. Solvent Casting

**Figure 2:**
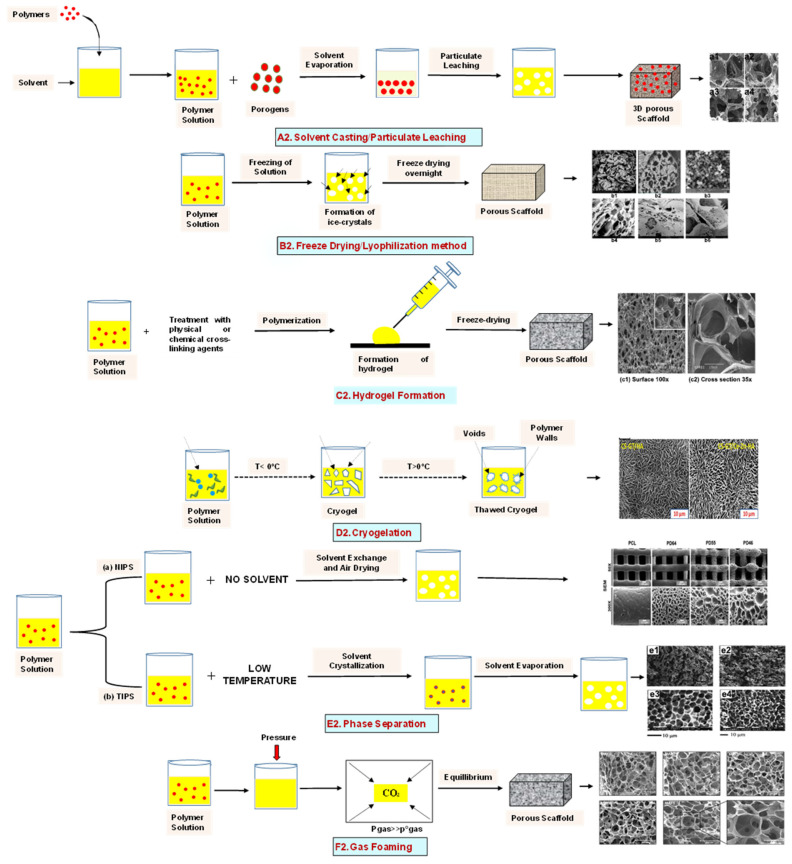
Schematic representation of traditional fabrication methods and microscopic images. (**A2**) Solvent casting and its microscopic image showing (**a1**) salt leached, (**a2**) salt-PEG 200 leached, (**a3**) salt-PEG 600 leached, and (**a4**) salt-PEG 1000 leached PCL scaffolds. Reprinted with permission from Ref. [43]. Copyright 2014, John Wiley & Sons, Ltd (**B2**) Freeze-drying method. Reprinted with permission from Ref. [44]. Copyright 2006, Elsevier Ltd.; and its SEM results of (**b1**) pure CS, (**b2**) pure ZN (zein), (**b3**) pure nHAp (nanohydroxyapatite), (**b4**) composite COM-10 i.e. ZN:CS:nHAp in 45:45:10, (**b5**) composite COM-15 i.e. ZN:CS:nHAp in 45:45:15, (**b6**) composite COM-20 i.e. ZN:CS:nHAp in 45:45:20. Reprinted with permission from Ref. [45]. Copyright 2018, Elsevier Ltd. (**C2**) Hydrogel formation and its (**c1**) surface and (**c2**) cross-sectional view. Reprinted with permission from Ref. [46]. Copyright 2019, Elsevier Ltd. (**D2**) Cryogel. Reprinted with permission from Ref. [47]. Copyright 2013, Korean Academy of Periodontology; FE-SEM images of developed biocomposites (chitosan-gelatin-hydroxyapatite and chitosan-gelatin-zinc doped hydroxyapatite). Reprinted with permission from Ref. [48]. Copyright 2020, Elsevier Ltd. (**E2**) Phase separation (**a**) NIPS based scaffolds and surface view of scaffolds (PCL, PD64 (PCL/DMSO in 60:40), PD55 (PCL/DMSO in 50:50) and PD46 (PCL/DMSO in 40:60) with its average pore size of the developed scaffolds. Reprinted with permission from Ref. [49]. Copyright 2020, RSC, and (**b**) TIPS and its SEM images of (**e1**) PLLA/HA scaffold in 50:50, (**e2**) PLGA/HA scaffold (50:50) with 2.5% (*w*/*v*) concentration of polymer and −18 °C quenching temperature (**e3**) PLLA scaffold and (**e4**) PLGA scaffold with 10% (*w*/*v*) polymer concentration; quenching condition: liquid nitrogen; volume ratio of dioxane and water at 87:13. Reprinted with permission from Ref. [50]. Copyright 2014, John Wiley & Sons, Ltd. (**F2**) Gas Foaming and its SEM micrograph representation of macroporous alginate foams showing well separated pores, among which MAF5 (Sr2+/Ca2+ molar ratio at 0:100) depicts interconnected porous structure. Reprinted with permission from Ref. [51]. Copyright 2018, Elsevier Ltd.; Reprinted with permission from Ref. [52] Copyright 2010, World Scientific (all adapted from [53,54]).

Solvent casting is an easy method for the fabrication of polymer scaffolds with porous networks. In this technique, the polymer of choice is liquefied in an organic solvent. The porogen, such as sodium chloride (NaCl), is added to the solution, resulting in the pore’s creation by forming a polymer–porogen network. The solvent evaporates, leaving behind the hardened polymer scaffold (Figure 2(A2)). Polymer scaffolds of controlled porosity can be fabricated by this method. However, the control of the pore shape and pore interconnectivity limits its application [14,15].

### 2.2. Freeze-Drying Method

The freeze-drying/lyophilization technique is a versatile method for the fabrication of polymeric porous scaffolds without the requirement of porogens. In this method, a water-based polymer solution is frozen, leading to ice crystal formation. The polymer aggregation takes place in the interstitial spaces around the ice crystals [16]. The removal of the remaining solvent subsequently takes place by applying pressure through the vacuum at a level lower than the frozen solvent’s equilibrium vapor pressure, resulting in the production of dry interconnecting porous scaffolds by complete solvent sublimation (Figure 2(B2)) [17,18,19,20,21]. Furthermore, the remaining water residue, which was not solidified before, is removed by the secondary drying process through the desorption method [22]. The direction of freezing has a huge impact on the pore morphology of the scaffolds. Directional freezing is defined as the aligned directional freezing of ice crystals in one direction from low to high temperature ends to create freeze-dried scaffolds with unidirectional oriented pores. This technique involves the fabrication of a broad range of porous structures and particulate materials using polymers in emulsion, solution, and colloidal suspension forms [23]. Emulsification freeze-drying is also a scaffold fabrication process employing polymers/ceramics dissolved in a solvent and subsequent mixing with water to obtain an emulsion. The blended emulsion solution is kept in a mold and frozen before the separation of the two phases. The resulting frozen emulsion is lyophilized to obtain a porous scaffold by the removal of the solvent and the dispersed water [24]. It is used to fabricate a wide range of polymer-based scaffolds without porogens, but the small pore size of the scaffold and the irregular porosity limit its application [25].

### 2.3. Hydrogels

Hydrogels are a highly hydrophilic 3D cross-linked networks that are used for the fabrication of extracellular matrix (ECM)-based scaffolds (Figure 2(C2)). They have a unique property to absorb 1000 times their original weight without mixing in an aqueous environment [26]. They are used in the tissue engineering field due to their biocompatibility and tunability as a tissue structure [27,28]. Natural polymers such as collagen, gelatin, chitosan, agarose, alginate, and hyaluronate are utilized for hydrogel synthesis, as they provide an ECM environment, but a lack of mechanical strength and uncontrollable degradation as well as high immunogenicity limit their application [19,29,30]. Therefore, they are blended with synthetic polymers such as poly (vinyl alcohol) (PVA), poly (2-hydroxyethyl methacrylate) (PHEMA), and poly- (ethylene oxide) (PEO) to overcome these drawbacks [31].

### 2.4. Cryogel Formation

The cryogelation process was first carried out in the 1970s. It involves the gelation of a polymeric solution at a subfreezing temperature, leading to the formation of cross-linked polymers surrounded by frozen water crystals. The cross-linked polymeric crystals are thawed to obtain a cryogel, which is an interconnected macroporous network of polymeric material (Figure 2(D2)). By this technique, one can control the porosity of scaffolds. Moreover, the alteration of the porosity and mechanical behavior of the scaffold can be amended by the incorporation of composite fillers and fibers in the polymer solution [32,33]. It is used in tissue engineering, bioseparations, biosensors, cell culture, cell delivery, drug delivery, and cancer immunotherapy, as there is no possibility of a thermal degradation of drugs and growth factors by this technique. 

### 2.5. Phase Separation Method

The fabrication of a porous polymeric scaffold takes place by the phase separation method by relying on the alteration in thermal energy involving the de-mixing of a desirable polymer in two immiscible solvents. The solutions of polymer-like poly(l-lactic acid) (PLLA) become thermodynamically unstable at low temperatures. When they are subjected to elevated temperature, the saturation of the solution takes place and results in separation into a polymer-rich phase and a solvent-rich phase. They are subjected to a high temperature, followed by quenching. At this point, liquid–liquid phase separation occurs. The polymer-rich phase results in solidification or precipitation to obtain a highly porous structure in the polymer matrix, whereas the solvent-rich phase is eliminated by extraction, sublimation, or evaporation (Figure 2(E2)) [25]. The phase separation method is divided into non-solvent-induced phase separation (NIPs) and thermally induced phase separation (TIPs).

NIPs: In this technique, the polymer is liquefied with a solvent, cast, and exposed to air for a relatively short duration of time. It is then immersed in a bath containing a nonsolvent solution, where polymer precipitation takes place due to the contact of the polymer solution with a nonsolvent. It leads to the formation of two phases: the polymer-rich phase and the polymer-poor phase. The solidification of the polymer-rich phase forms a porous structure (Figure 2(E2(a))) [34,35]. NIPs-generated scaffolds have limited use in tissue engineering applications.

TIPs: In the TIPs process, the homogenous polymer solution is prepared at an elevated temperature, followed by quenching to induce phase separation. Upon cooling, the homogenous polymer solution separates into polymer-poor and polymer-rich phases. Upon solidifying, the scaffold matrix is created by the polymer-rich phase, whereas the solvent is removed in the polymer-poor phase, creating pores (Figure 2(E2(b))). TIPs is classified into solid–liquid (S-L) phase separation and liquid–liquid (L-L) phase separation. In solid–liquid phase separation, the crystallization of the solvent takes place by lowering the temperature. When these solvent crystals are removed, pore formation takes place. L-L phase separation is characterized by the coexistence of both the polymer-rich and polymer-poor phases. The porous structure is formed due to de-mixing at specific temperatures and concentrations [22,36]. The phase separation takes place by binodal de-mixing and/or spinodal decomposition. Binodal de-mixing forms a porous scaffold with a poor interconnected network, whereas spinodal decomposition results in a well-interconnected network. 

### 2.6. Gas Foaming Method

Gas foaming involves bubble formation in the polymer solution. The polymer solution is compressed into a solid form and pressurized by a gas foaming agent, such as water (H_2_O), fluoroform, nitrogen (N_2_), or carbon dioxide (CO_2_), until saturation takes place [37,38]. The formation of gas bubbles in the range of 100 and 500 µm takes place by this method (Figure 2(F2)) [39,40]. The size of the pores can be controlled by adjusting the mixing ratios of both the polymers and the foaming agents. Moreover, gas foaming is initiated by a reaction during the blending process of two chemicals, leading to the release of N_2_ gas. This gas release leads to foam formation with a highly porous network [41]. The main advantage of gas foaming is the use of nontoxic solvents, but poor pore interconnectivity and a nonporous external surface due to bubble formation limit its application [42].

## 3. Electrohydrodynamic Technique

The electrohydrodynamic technique is a fabrication method in which an electrically charged fluid is fed in a syringe, which after being subjected to an electric field, comes out of the nozzle and is collected on an oppositely charged collector. It comprises a high-voltage power setup, a syringe with a metallic syringe, and an oppositely charged collector. The liquid polymer solution is fed in a syringe, and it forms droplets at the needle tip due to surface tension. When a high voltage is applied, electrostatic repulsion overcomes the surface tension, and the fluid comes out of the syringe as a ‘Taylor cone’ [55]. Finally, it forms a charged jet that is collected on a plate, resulting in the formation of nanostructures/microstructures [55]. Concentration is a key factor that governs the morphology of the obtained structure [56]. Low-concentration solutions result in spherical particles. With an increase in concentration, the formation of beads with fibers takes place. A solution above the critical concentration results in uniform fibers. Helix-shaped microribbons are formed at very high solution concentrations. Electrohydrodynamic methods, including electrospraying and electrospinning, have become a research hotspot to produce fibers in the micro- or nano-range.

### 3.1. Electrospray Technique

**Figure 3:**
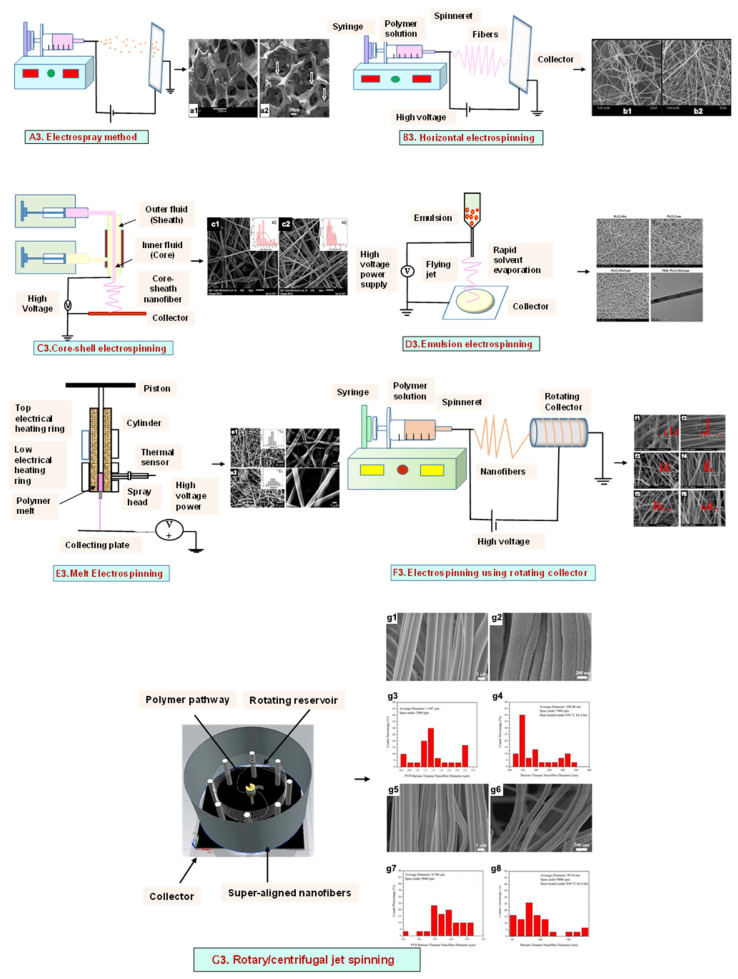
Illustration of electrohydrodynamic techniques and their scanning electron microscope images. (**A3**) Electrospray method. Reprinted with permission from Ref. [80]. Copyright 2018, Taylor & Francis; and its macroporous ZrO2 foams structure formed by the combination of (**a1**) electrospraying and (**a2**) slurry dipping, respectively. Reprinted with permission from Ref. [81]. Copyright 2006, John Wiley & Sons, Ltd. (**B3**) Horizontal electrospinning. Reprinted with permission from Ref. [82]. Copyright 2016, Penerbit UTM Press, Universiti Teknologi Malaysia; and SEM micrographs of (**b1**) cellulose acetate (CA) scaffolds (9%) and (**b2**) regenerated cellulose scaffold (9%). Reprinted with permission from Ref. [83]. Copyright 2019, Elsevier Ltd. (**C3**) Core-shell electrospinning. Reprinted with permission from Ref. [84]. Copyright 2019, John Wiley & Sons, Ltd; SEM morphology of core-shell PCL-PLA/HA electrospun fibres at (**c1**) 2:3 and (**c2**) 3:3 core:shell flow rate ratio (marker bars at 20 μm) including (**c1.1**) and (**c2.1**) depicting the histograms of the fibre diameters at 2:3 and 3:3 flow rates. Reprinted with permission from Ref. [85]. Copyright 2019, IOP Sceince (**D3**) Emulsion electrospinning. Reprinted with permission from Ref. [86]. Copyright 2018, Elsevier Ltd; FE-SEM images of: (**A**) PLCL/HA (poly (L-lactic acid-co-ϵ-caprolactone)/hydroxyapatite), (**B**) PLCL/lam ((poly(L-lactic acid-co-ϵ-caprolactone)/hydroxyapatite/laminin), and (**C**) PLCL/HA/Lam nanofibers; and (D) TEM images of HA loaded PLCL/HA/Lam nanofibers. Reprinted with permission from Ref. [87]. Copyright 2013, Taylor & Francis (**E3**) Melt electrospinning. Reprinted with permission from Ref. [88], Copyright 2012, doiSerbia; SEM images of (**e1**) solvent-based electrospun fibers and (e2) melt-based electrospun fibers. Reprinted with permission from Ref. [89]. Copyright 2013, RSC (**F3**) Electrospinning using rotating collector Reprinted with permission from Ref. [90]. Copyright 2019, MDPI; FE-SEM of (**f1**–**f3**) randomly-oriented and aligned (**f4**–**f6**) electrospun nanofibers at weight ratios of PLGA/gelatin of (**f1**,**f4**) 10: 0, (**f2**,**f5**) 9:1, (**f3**,**f6**) 7:3. Reprinted with permission from Ref. [91]. Copyright 2010, Elsevier Ltd. (**G3**) Rotary/centrifugal jet spinning. Reprinted with permission from Ref. [70]. Copyright 2017, John Wiley & Sons; Morphology and distribution of fiber diameter obtained before and after annealing and by spinning at two different speeds 7000 rpm and 9000 rpm, respectively as depicted in (**g1**–**g4**) and (**g6**–**g8**). (**g1**) Polyvinylpyrrolidone-barium titanate (PVP–BaTiO3) fiber spun at 7000 rpm, (**g2**) BaTiO3 nanofiber calcined at 850 °C spun at 7000 rpm, (**g3**) fiber diameter distribution of PVP–BaTiO3 spun at 7000 rpm, (**g4**) BaTiO3 fiber diameter distribution annealed at 850 °C spun at 7000 rpm, (**g5**) PVP–BaTiO3) fiber spun at 9000 rpm, (**g6**) BaTiO3 nanofiber calcined at 850 °C spun at 9000 rpm, (**g7**) fiber diameter distribution of PVP–BaTiO3 spun at 9000 rpm, (**g8**) fiber diameter distribution of BaTiO3 annealed at 850 °C spun at 9000 rpm. Reprinted with permission from Ref. [79]. Copyright 2014, Elsevier Ltd.

The electrospray method results in droplet formation, instead of the jet in electrospinning, due to the interactions of bulk and surface electrohydrodynamic forces (Figure 3(A3)). The deposition of jet fragments on the collector attains a spherical shape due to surface tension. It depends on various factors such as conductivity [57], voltage [58], and the surface tension [59] of the polymer solution to be sprayed. Moreover, when the density [59], flow rate [60], and viscosity [61] of the sprayed polymer solution increase, the particle diameter increases. The electrospraying technique was recently applied in drug delivery applications. 

### 3.2. Electrospinning

Electrospinning is a process that employs a high voltage to fabricate micro/nanostructure fibers using a polymer or a molten liquid [62]. In comparison with other conventional scaffold methods, electrospun nanofibers are regarded as an ideal tissue engineering scaffold due to their complex interface topology, large surface area, and ease of functionalization. There are a few parameters that affect the size of the nanofibers, such as the polymer’s molecular weight, the conductivity and viscosity of the solution, the surface tension, the flow rate, the voltage, and distance between the nozzle tip and the collector. In the BTE field, electrospun nanofibers possess an open structure, providing a biomimetic environment to support cell connections in all directions. The polymer solution is fed in the syringe tube. The needle serves as the positive terminal, whereas a metal collector serves as a negative collector [63]. When the intensity of the electric field increases, the electrostatic repulsion overcomes the surface tension, and the polymer solution is ejected as a ‘Taylor cone’. There is instability in the discharged polymer jet, allowing the polymer to be very long. The solvent dissolved in the polymers evaporates, resulting in the drying of the polymer in the jet.

#### 3.2.1. Horizontal Electrospinning

Horizontal Electrospinning is a traditional and versatile method of electrospinning. In this electrospinning method, the syringe containing the polymer solution is placed horizontal/parallel to the platform, while the collector is placed vertically to collect the fiber (Figure 3(B3)). With the application of a high voltage to the polymer solution, due to charge repulsion there is the generation of a force in the charged polymer solution that overcomes the surface tension. This leads to the formation of a conical-shaped ‘Taylor cone’ [64] that stretches to form a stable jet that is collected as nonpatterned nanofibers on the oppositely charged collector [64]. It is the most frequently used electrospinning method, but the lack of tensile strength of the nonpatterned nanofiber and the wide range of fiber thickness limits its application. To minimize this, various modified electrospinning techniques with better properties are preferred [65].

#### 3.2.2. Core–Shell Electrospinning

Coaxial electrospinning/core–shell electrospinning employs two different solutions ejected by a coaxial nozzle with a core–shell structure obtaining encapsulating material [66,67]. It comprises two spinnerets of dissimilar sizes, i.e., the smaller inner diameter forming the core solution and the larger outer diameter forming the shell solution (Figure 3(C3)). Both the core and the shell solutions are kept in separate reservoirs. The generation of core–shell nanofiber takes place after the ejection of solutions through the coaxial nozzle [68]. Oil is often used as a temporary material during the postspinning process, as it is relatively easy to eliminate it from high-molecular-weight solutions [69]. It is widely used for tissue engineering applications due to its controlled release of drugs or growth factors. However, it does not fulfill the requirement of greater porosity on the shell surface of the nanofibers [70].

#### 3.2.3. Emulsion Electrospinning

Emulsion electrospinning is a quick and easy method to create micro- and nanofibers with a core–shell configuration. Lots of polymers are used for emulsion electrospinning due to their abundance, comprising biopolymers (e.g., polysaccharides and proteins) and biocompatible polymers (e.g., PEO, PVA, and poly (ε-caprolactone) (PCL)). These emulsion-based nanofibers using these polymers have attracted researchers’ attention in biomedical and various other fields due to their biocompatibility, low toxicity, and biodegradability. The fabricated electrospun micro- and nanofibers possess outstanding physicochemical properties and mimic the ECM, supporting cell adhesion and nutrition transfer. To date, emulsion-electrospun nanofibers have lots of applications in pharmaceutical and biomedical areas [71], incorporating biomolecules or hydrophilic drugs in water-in-oil emulsions to attain the sustained release of the drugs/biomolecules (Figure 3(D3)) [72].

#### 3.2.4. Melt Electrospinning

Melt electrospinning is an ecofriendly, solvent-free method that has attracted various biomedical researchers. The electrospun fibers obtained by melt electrospinning are in the range of a few microns and are designed to attain 3D structural forms by mutual support. This matrix form is advantageous for cell adhesion, movement, growth, and maturity (Figure 3(E3)). Dalton, in 2006, was the first to propose melt-electrospun fibers of 1–2 µm using a blend of PEO-block-PCL with PCL [73]. Later, in 2008, the controlled graphics of melt electrospinning in tissue engineering applications was explained by Dalton [74]. It offers the advantages of scalability, but the large diameters of electrospun fibers limit its application [75].

#### 3.2.5. Rotating Collector Electrospinning

In the tissue engineering field, the orientation of fibers is an important factor that helps in cell alignment and ECM deposition [76,77]. Moreover, fiber alignment helps in neotissue formation with better mechanical properties [76,78]. To obtain an aligned fiber, a rotating mandrel with a high rotational speed is used as a collector. Apart from this, researchers also put their efforts into improving the fiber direction through the use of a better collector design (Figure 3(F3)). It produces aligned nanofibers that help in bone regeneration, but less mass production of nanofibers limits its application. 

#### 3.2.6. Rotary/Centrifugal Jet Spinning 

Rotatory jet spinning is a low-cost and versatile technique to fabricate highly aligned electrospun fibers using protein–polymer materials. It produces anisotropic nanofibrous scaffolds at a high production rate without the use of a high-voltage electric field, which is the main drawback of conventional electrospinning methods [70]. In this method, the polymer solution is persistently supplied in a chamber, and a centrifugal force is applied. When the rotatory force exceeds the capillary force, the spinning solution in the form of a jet is ejected from the chamber (Figure 3(G3)). The ejection of the solution results in elongated aligned nanofibers due to various factors such as the centrifugal force, the angular speed of the rotating chamber, the evaporation of solvents in the polymer solution, and the viscosity of the solutions [79]. Although it produces electrospun nanofibers at a low power consumption, to date it is limited to a few polymers [75].

## 4. Additive Manufacturing (AM) Techniques 

The AM method entails a variety of fabrication techniques in which 3D objects are constructed by the addition and processing of materials in a sequential manner or a layer- by-layer fashion via commercial computer-aided design (CAD) tools [92,93]. Bone scaffolds with precisely predefined internal and external architectures can be created using AM’s special set of capabilities. Some widely used AM techniques include 3D printing, fused deposition modeling (FDM), and selective laser sintering (SLS).

### 4.1. Three-Dimensional Printing

Three-dimensional printing is a fabrication method that uses ceramics, powders, plastics, metals, liquids, or even living cells as bioink to produce a 3D construct by adding them successively in a layer-by-layer form. Viscosity, gelation, and cross-linking are the basic properties of bioink that affect the quality of printed objects, morphology, and protection during the printing process, which affect cell attachment, viability, and the proliferation of cells [94]. Finally, a 3D model is formed by the solidification of bioink under a 3D modeling program attached to a computer [95].

#### 4.1.1. Extrusion-Based Bioprinting

**Figure 4:**
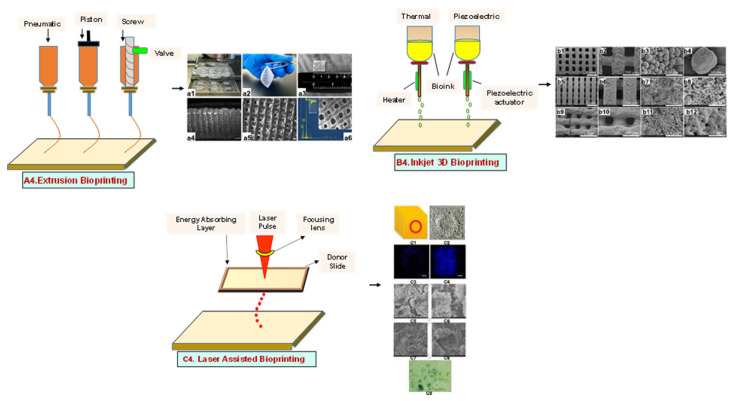
Schematic representation of different types of 3D printing methods and their microscopic images: (**A4**) Extrusion method. (**a1**) The 3D printing procedure, (**a2**,**a3**) Camera images showing 3D printed hydrogel construct. (**a4**,**a5**) High magnification images of the surface of the construct showing pores and struts morphology (scale bars at 1000 and 500 µm, respectively). (**a6**) The elemental composition of the surface of the mesoporous silica-calcia nanoparticles containing hydrogel supported by a SEM image of the construct. Reprinted with permission from Ref. [107]. Copyright 2021, Elsevier Ltd. (**B4**) inkjet 3D bioprinting. SEM microstructure displaying spherical powder sintered scaffolds (**b1**–**b4**), in addition to micropores on air jet milling powders sintered scaffolds surface (**b5**–**b8**), nano-sized grains sintered scaffolds showing many cracks (**b9**–**b12**). Reprinted with permission from Ref. [108], Copyright 2018, Elsevier Ltd. (**C4**) laser-assisted bioprinting (**c1**) Design of experiment, (**c2**) Optical microscopy at day 0 (bar = 150 μm). (**c3**) Fluorescence microscopy showing cell migration and proliferation on day 3 (bar = 200 μm). (**c4**) Fluorescence microscopy showing a complete covering of the initial nHA pattern at day 6 (bar = 200 μm). (**c5**,**c6**) Scanning electron microscopy of the nHA surface. HOP cells spread onto the material on day 3. (**c7**,**c8**) Scanning electron microscopy of the nHA surface. HOP cells spread onto the material on day 6. (**c9**) ALP activity assay showing that HOPs maintain their osteoblastic phenotype at day 6. Reprinted with permission from Ref. [109] Copyright 2011, IOPscience (all adapted from [110]).

An extrusion-based printing system assists the extrusion of biomaterials through a micronozzle without any heating process. In this method, the deposition of biomaterials mixed with cells on the stationary print bed in the XY plane is performed, followed by Z-axis in a layer-by-layer fashion to create a 3D structure. The viscous hydrogels utilize piston, screw, or pneumatic pressures as the driving force for deposition on a stationary substrate (Figure 4(A4)) [96]. This method is used to fabricate porous scaffolds that help in cell proliferation. A high cell density is printed rapidly by the extrusion-based method. However, the cell viability is affected, and cell distortion takes place due to shear stress or the applied pressure [97].

#### 4.1.2. Inkjet Bioprinting

Inkjet bioprinting is a droplet-based bioprinting method used to generate a 3D model by placing biomaterials on the substrate in a layer-by-layer fashion. Inkjet bioprinters are categorized into thermal or piezoelectric types [98,99]. A thermal inkjet bioprinter heats the biomaterials locally with a voltage pulse by a thermal actuator, resulting in the formation of a small vapor bubble. This provides the pressure pulse to overcome the surface tension and pushes the droplet via the nozzle (Figure 4(B4)). In piezoelectric inkjet bioprinting, voltage pulses on both sides of the piezoelectric actuator compress the piezoelectric element and change the liquid volume. This forces the biomaterials to squeeze out of the nozzle and drop onto the substrate. It is a widely accepted method, as it is readily available, fast, and economic. However, a lack of precision affecting the droplet size and placement limits its applications [100]. 

#### 4.1.3. Laser-Assisted Bioprinting

Laser-assisted bioprinting is a nozzle-free, noncontact technique that uses a laser beam as energy to accurately deposit a high-resolution biomaterial on a solid substrate [101]. It consists of a light source (laser), a ribbon coated with a gold or titanium layer onto which the biomaterial is spread, and a substrate. The working procedure begins with the evaporation of the biomaterial and droplet formation when the laser strikes the ribbon (Figure 4(C4)). A high-pressure bubble forms due to evaporation, causing droplets to accumulate on the substrate [102,103,104]. This technique is repeated until a functional 3D construct is formed. This bioprinting offers a high degree of precision and resolution, making it suitable for bioprinting DNA, cell arrays, and micropatterned peptides [102,105]. However, its applicability is limited by its low cell viability and time-consuming process [106]. 

### 4.2. Fused Deposition Modeling 

**Figure 5:**
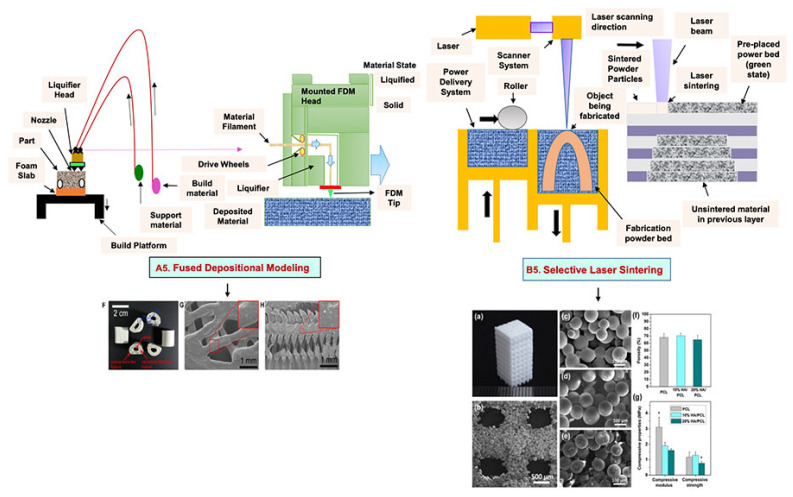
Schematic illustration of (**A5**) FDM. Reprinted with permission from Ref. [53]. Copyright 2020, Elsevier Ltd.; (**F**) The developed PCL/HA 3D artificial bones. (**G**) Surface view of PCL/HA 3D artificial bone, in which upper-right image is magnified. (**H**) Cross-sectional view of PCL/HA 3D artificial bones, in which the upper-right image is magnified from the corresponding area. Reprinted with permission from Ref. [114]. Copyright 2014, ACS Publications and (**B5**) SLS (adapted from [53]); (**a**) The developed sintered scaffold of cuboid-shaped morphology and highly ordered porous structure. (**b**) SEM image showed the detailed morphology of pores in a representative 10% HA/PCL scaffold. (**c**–**e**) SEM micrographs verified the microspheres were well connected via laser sintering in PCL scaffolds (**c**), 10% HA/PCL (**d**) and 20% HA/PCL (**e**). (**f**) The porosity analysis (**g**) mechanical properties of the scaffolds. All data represented the mean ± SD; n = 5, * *p* < 0.05 (data compared with other two groups). Reprinted with permission from Ref. [115]. Copyright 2015, Elsevier Ltd.

FDM is a solvent-free fabrication method involving an extrusion-based 3D additive manufacturing technique. It fabricates a scaffold with better dimension precision and product quality in less time [111]. In this method, the thermoplastic in a thin layer is deposited by a temperature-controlled extruder providing support in the layer-by-layer form [112]. The resolution of the FDM construct is affected by various factors such as the nozzle diameter and the type of polymer material. It fabricates highly porous scaffold structures with controlled porosity (Figure 5(A5)). It is used to fabricate surgical guides, implants, and prostheses. However, direct cell printing by the FDM process is not possible due to the degradation of cells by high temperatures and unfavorable pH environments. 

### 4.3. Selective Laser Sintering 

SLS employs a high-power laser beam to increase the temperature of a material, such as plastic, metal, ceramic, or glass powder, for the fusion of the powder in a layer-by-layer form without melting to attain a 3D construct (Figure 5(B5)) [113]. This technique was first developed by the University of Texas in 1986. Lots of polymers are fabricated by this method, such as PLLA, PVA, polyamide (PA), polyether ether ketone (PEEK), and PCL. However, due to the increased temperatures, the problem of loading viable cells and biomaterials directly into the scaffold is the limitation of this technique.

### 4.4. Binder Jetting

**Figure 6:**
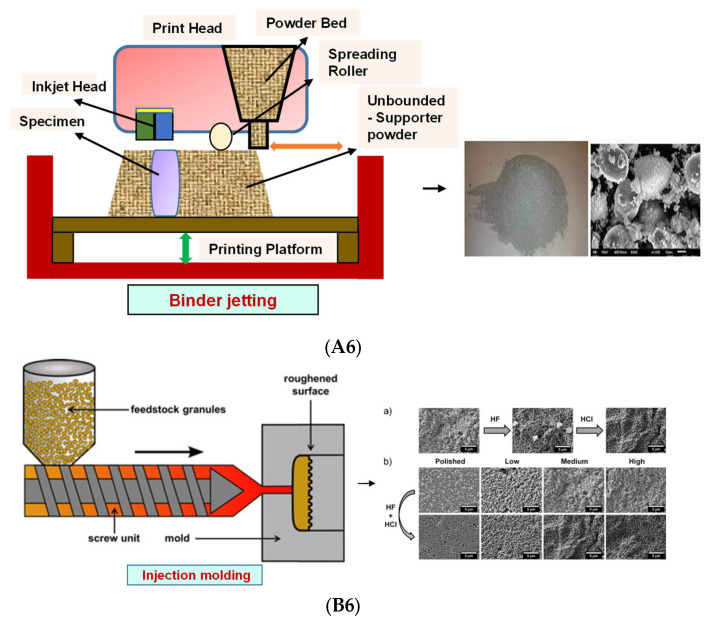
(**A6**) Schematic illustration and scanning electron microscope images of BJ. Reprinted with permission from Ref. [125], Copyright 2021, CSI; SS316- Tricalcium phosphate (left) and its morphology (right). Reprinted with permission from Ref. [126], Copyright 2017, Conference Reviewed Paper. (**B6**) Schematic representation of injection molding and (**a**) microscopic observations of the surface of zirconia toughened alumina at the various steps of the selective etching process, demonstrating the formation of fluoride precipitates during hydrofluoric acid etching and their subsequent removal in HCl; (**b**) FE-SEM observations of the surface of injection molded samples with different surface topographies before and after selective etching. Low, Medium and High surfaces attained from increasingly rough molds. Polished entitles the surface of samples that were polished after sintering. Scale bars: 5 μm. Reprinted with permission from Ref. [127], Copyright 2016, Elsevier Ltd.

Binder jetting (BJ)/powder-based 3D printing is a flexible approach for fabricating bone-tissue-engineered scaffolds that involves combining loose powder materials with a liquid binding agent to generate a 3D structure with considerably more control over geometry [116,117]. The binder should be selectively sprayed onto the powder region bed by the binder delivery system to obtain the solid entity. It provides accuracy and flexibility to the matrix with intricate geometry (Figure 6(A6)). However, the lack of mechanical strength of the scaffold and difficulties in loading medications and other biological factors directly to the 3D matrix limit its application [118].

### 4.5. Injection Molding

Injection molding is among the most prevalent polymeric product fabrication techniques used for research in BTE applications. The modification of the glass transition temperature by the combination of various polymeric types, which may change the mechanical features to desired parameters, makes this technique easily adaptable (Figure 6(B6)) [119]. PLA and PCL are the most extensively utilized polymers as potential scaffold materials due to their biocompatibility and accessible operating conditions [120,121]. Injection molding, on the other hand, has a significant disadvantage in that it is difficult to manufacture porous surfaces inside the monolithic final product that results from moderately homogenous solidification, which affects cell proliferation, migration, and regeneration [122]. This disadvantage is overcome by the use of porogens, which showed promising results in the generation of better pore development [123]. Porosity is also induced in injection-molded biomaterials by the use of microcellular injection molding due to its unpredicted level of precision without organic solvents and any environmental complications [124].

## 5. Photolithography

Photolithography is a top-down approach involving the transfer of a geometric pattern from a photomask to a light-sensitive photoresist on a substrate. For the preparation of the photoresist coating, the cleaning of a silicon wafer as a substrate is an important step to improve the efficiency of photolithography [128]. The baking of the wafer prevents readsorption [129]. The cleaned wafer is further subjected to photoresist coating after the deposition of ultraviolet (UV) radiation, followed by the prebake (soft bake) [130]. The final step includes exposure to UV light through a photomask on a substrate and the transfer of a pattern onto the photoresist [131]. It is used to pattern biomaterials such as cells, proteins, and ECM, but maintenance and cleanliness for the proper functioning of this instrument is a drawback of this method.

### 5.1. Stereolithography Technique

**Figure 7:**
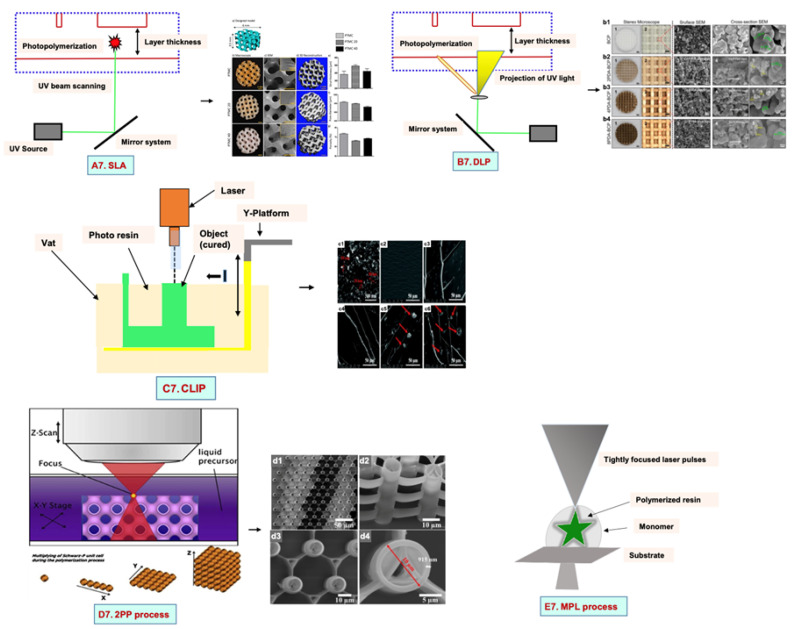
Mechanisms of various types of photolithography with microscopic images: (**A7**) SLA. poly (trimethylene carbonate) microporous (PTMC) scaffolds with 20 and 40% of hydroxyapatite (HA) (**a**) Model design (**b**) macroscopic and (**c**) microscopic SEM images of all scaffolds (**d**) 3D architecture (**e**) strut thickness (**f**) pore diameter (**g**) porosity of all SLA fabricated scaffolds. Reprinted with permission from Ref. [141], Copyright 2022, Frontiers (**B7**) DLP. Surface morphology, surface and cross-sectional view of all scaffolds with PDA (polydopamine) modification dipped in Tris-HCL at concentration of 2 mg/mL, 4 mg/mL and 8 mg/mL. (**b1**) Biphasic calcium phosphate (BCP) (**b2**) 2PDA-BCP. (**b3**) 4PDA-BCP. (**b4**) 8PDA-BCP. Deposition of Ca-P/PDA is showed by white stars, white arrows designate the PDA layer and amorphous Ca-P nanoparticles. Green arrows illustrated by green arrows. the size of newly formed amorphous Ca-P nanoparticles is showed by yellow circle. Reprinted with permission from Ref. [142], Copyright 2020, Frontiers ((**A7**,**B7**) adapted from [143]), (**C7**) CLIP. Reprinted with permission from Ref. [144], FE-SEM images of (**c1**) Pristine nHA and (**c2**–**c6**) Fractured surface of of poly(ethyleneglycol)diacrylate containing 0 wt%, 0.5 wt%, 1.0 wt%, 1.5 wt%, and 2.0 wt% n-HA. Copyright 2021, RSC; Reprinted with permission from Ref. [145], Copyright 2018, IOPsceince (**D7**) the 2PP process. (**d1**) Large area over-view (**d2**) side-view tilted at 30 °C (**d3**) Top view and (**d4**) closer top view of IP-L780 photopolymer. Reprinted with permission from Ref. [146], Copyright 2008, Taylor & Francis; Reprinted with permission from Ref. [147], Copyright 2019, ACS and (**E7**) the MPL process. Reprinted with permission from Ref. [148], Copyright 2008, Elsevier Ltd.

Stereolithography (SLA) is a vat-based printing method that is fast and has excellent resolution with improved cell viability. In this method, the liquid-based biomaterial is continuously exposed to a laser beam (UV or visible light) to solidify it. In response to incoming light, a photoinitiator (PI) molecule in the resin triggers the chemical polymerization reaction locally, which results in curing only in the exposed portions. This leads to the development of the first layer, with the subsequent application of a fresh resin film, which thereby are irradiated and cured, resulting in the generation of a solidified photosensitive biomaterial in a layer-by-layer manner (Figure 7(A7)).

### 5.2. Digital Light Processing

Digital light processing (DLP) is a better-quality SLA that uses UV or blue light projections. The fast-processing speed and better resolution make it superior to SLA. In this method, the laser beam passes through the projector and is projected on a transparent plate. Therefore, the whole surface is cured [132]. By this technique, the researchers used a mixture of hydroxyapatite (HA) and a photosensitive resin (consisting of 98% methacrylate-based monomers and 2% photoinitiator) to print a bone scaffold (Figure 7(B7)) [132]. It has a high efficiency without the use of a laser or heating chamber, but the use of photosensitive resins that are cytotoxic limits its applications [132].

### 5.3. Continuous Liquid Interface Production/Digital Light Synthesis

Continuous liquid interface production/digital light synthesis (CLIP) is characterized by a continuous resin flow that allows for faster printing and a smoother surface. There is no pause after each layer of the structure. Properties such as accuracy, flexibility, and speed have attracted various researchers to produce 3D models with very fast speeds (Figure 7(C7)).

### 5.4. Two-Photon Polymerization

Two-photon polymerization (2PP) differs significantly from the previous techniques. The mechanism of two-photon absorption is used to create microstructures and nanostructures in polymer solutions [133]. A femtosecond pulsed laser beam passes through the solution and polymerizes it by confining it to the focal point rather than the entire area (Figure 7(D7)). The scaffolds have no geometrical limitations because they are not fabricated layer by layer. This approach produces scaffolds that resemble ECM and support cell adhesion and growth [134,135].

### 5.5. Multiphoton Polymerization/Multiphoton Lithography

The multiphoton polymerization/multiphoton lithography (MPP/MPL) process requires a combination of multiphoton absorption (MPA), including a nonlinear chemical or physical reaction of the material to local photoexcitation, to create complex 3D structures [136,137,138]. An ultrafast pulsed laser beam focused in a completely transparent photopolymer causes MPA. Local polymerization begins due to the occurrence of MPA around the focal point. A focal spot within the total volume is scanned, followed by photopatterning of a high-resolution 3D structure [139,140]. The sample in the 3D structure is obtained as a replica of the photo pattern by immersing the sample into a solvent for the removal of unexposed material (Figure 7(E7)). Additionally, cells are printed without any external force. High cell viability is the advantage of this technique, but high costs and slow speeds limit its application.

## 6. Microsphere-Based Sintering Method

Microsphere-based scaffolds have attracted researchers’ attention due to their easy fabrication, physicochemical characteristics, and controlled morphology. There are various methods to fabricate microspheres, such as a solvent vapor treatment (dichloromethane) [149], heat sintering [150,151], a solvent/nonsolvent sintering method (acetone and ethanol treatment) [152,153], and a nonsolvent sintering technique (ethanol treatment) [154], requiring high temperatures and the use of organic solvents, which limits its application in pharmaceutical and biomedical applications [155]. Therefore, the CO_2_ sintering method is preferred to fabricate cells containing a matrix with high viability. In 2020, Gils Jose et al. performed in vitro and in vivo studies using sintered bone graft scaffolds made of nanohydroxyapatite and nanowhitlockite within poly (lactic-co-glycolic acid) (PLGA) microspheres [142].

## 7. Four-Dimensional Printing

**Figure 8:**
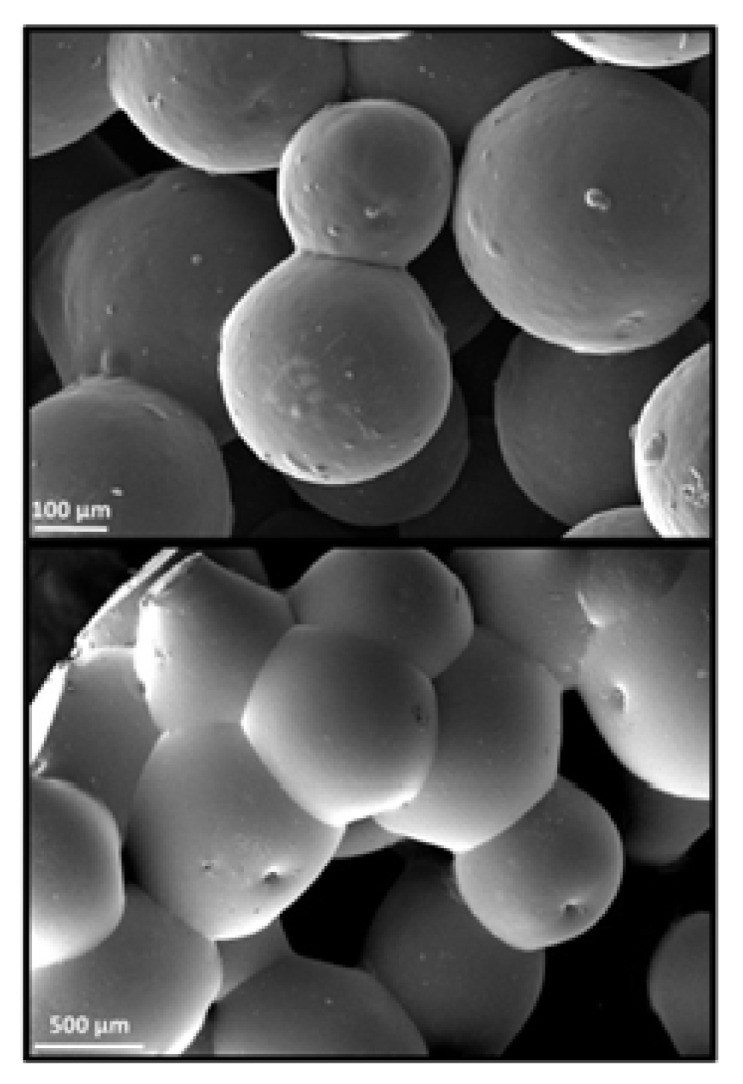
SEM images of microsphere based sintered scaffolds, using PCL/0.5%TNT (TiO2 Nanotube) at 100 µm and 500 µm. Reprinted with permission from Ref. [159], Copyright 2021, AIP publishing.

**Figure 9:**
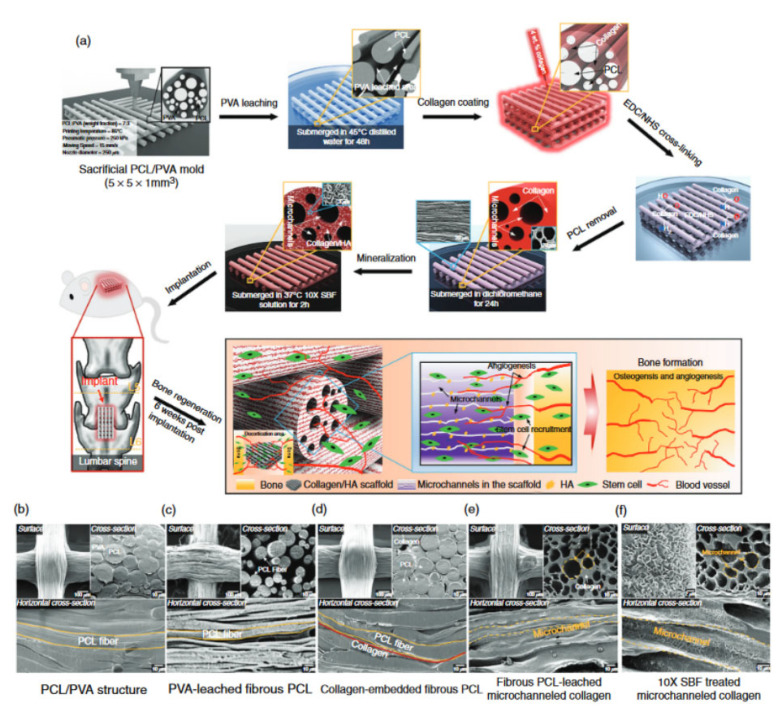
4D-printed scaffolds with its microscopic image. (**a**) Representation of development of mineralized, microchanneled collagen scaffold and in vivo assessment of osteogenesis and angiogenesis. SEM morphology of (**b**) PCL/PVA, (**c**) PVA-leached fibrous PCL, (**d**) collagen-embedded fibrous PCL, (**e**) collagen microchannels after leaching fibrous PCL, and (**f**) SBF-treated microchanneled collagen. Reprinted with permission from Ref. [160], Copyright 2020, Elsevier Ltd.

Four-dimensional printing is an advanced technology involving multimaterial printing, with its potential to alter over time, or a tailored material system that shifts from one shape to another. It may be utilized to create diverse 3D-shaped biologically live structures capable of dynamic configuration alterations in response to varied desired stimuli over time, utilizing stimuli-responsive materials to overcome the limitation of 3D bioprinting [156]. This method also helps to repair irregular-shaped bone defects through the use of shape-shifting scaffolds (Figure 8). In addition, it also helps to print material possessing stiffness- and morphology-shifting abilities [132]. Gao, et al. described 4D bioprinting (Figure 9) as a cell-filled 3D-printed construct that not only responds to internal stimuli or external stimuli but also to the maturation and functionalization of cells or tissues in 3D-printed constructions over time (i.e., the shape of the printed structures does not alter) [157]. All these criteria maintain the homeostasis and self-renewal of the fabricated biological constructs. However, there should be stability in the configuration and function of 4D-printed constructs before and after stimulation to provide the effective regeneration of the irregular bones. In addition, the mechanical properties of the produced scaffold should be altered by cross-linkers or by the use of stimuli-responsive materials [158].

## 8. Applications in Bone Tissue Engineering

### 8.1. Conventional Methods

In 2020, Huei-Yu Huang et al. developed a PCL/graphene 3D porous scaffold by a solvent casting/particulate-leaching method in which graphene improved the mechanical properties, hydrophobicity, cell attachment, and proliferation of MG-63 cells [161]. Another study reported the synthesis of boron-doped bioactive glass (B-BG) and blending in a collagen/gelatin solution to obtain collagen/gelatin/B-BG biocomposite scaffolds, which showed good porosity, osteoconductivity, and bioactivity [162,163]. The researchers utilized polyurethane (PU), polymethyl methacrylate (PMMA), and NaCl as a porogen to fabricate a 3D scaffold mimicking the bone marrow microenvironment [164]. Another study reported the alteration of surface topography using PCL and carbon nanotube (CNT)-reinforced PCL composites developed by the solvent casting method and electrospinning. Among these, solvent-casted scaffolds showed better mechanical strength, whereas electrospun films revealed enhanced viability [165].

**Figure 10:**
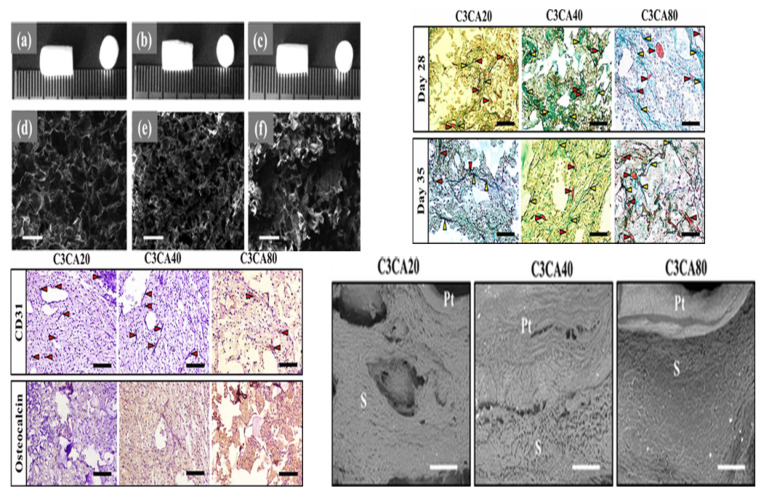
Fabrication of freeze-dried scaffolds via lyophilization technique using C3CA scaffolds. (**a**–**c**) Camera images and (**d**–**f**) cross-sectional view of C3CA scaffolds processed at −20, −40, and −80 °C depicting porous structures, and the pores were bigger in C3CA20 compared to those in C3CA40 and C3CA80. Scale bar: 100 μm. Masson’s trichrome staining of C3CA scaffolds sections after 28 and 35 days of implantation. Red arrows depicting blood vessels; yellow arrows depicting collagen deposition. Scale bar: 100 μm. Positive staining of CD31 verified the vascularization in the implants. The occurrence of mild staining of osteocalcin-positive cells designates the unconfined cells to osteoblastic lineage. Fibrous tissue in-growths into the pore spaces of implants as directed with no apparent alteration between peripheral tissues (Pt) and implant material (S). Scale bar: 500 μm. Reprinted with permission from Ref. [166], Copyright 2018, MDPI.

In 2021, Ganesan Priya et al. obtained freeze-dried carboxymethyl cellulose (C3CA) scaffolds, at −20, −40, and −80 °C (Figure 10). Scaffolds with larger pore sizes, i.e., 74 ± 4 µm, were achieved at −20 °C compared to −40 and −80 °C. In vitro results using the Saos-2 osteoblast cell line showed cytocompatibility, cell differentiation, and proliferation with vital mineralization. An in vivo analysis in a rat model using carboxymethyl cellulose (C3CA) scaffolds lyophilized at −40 °C showed new matrix tissue formation and vascularization within 28 days of implantation [166]. The fabrication of graphene-oxide-incorporated chitosan scaffolds by the freeze-dried method was reported, which showed excellent biocompatibility with the recovery of tissue architecture [167].

The researchers utilized the supercritical carbon dioxide (Sc-CO_2_) foaming technique to develop PLLA/poly (ethylene glycol) (PEG) porous scaffolds that possess good porosity and interconnected open pores with excellent biocompatibility, supporting bone tissue engineering [168]. Some studies combined the internal gelation technique with the gas foaming technique to obtain strontium cross-linked alginate foams of high porosity to promote bone regeneration [51].

The osteoconductive property of nano-hydroxyapatite (nHA) was examined on alginate–gelatin hydrogels. nHA reduced the swelling behavior and the mechanical strength. In vitro studies using MG63 cells showed improved proliferation and osteogenesis due to an increase in the concentration of gelatin with nHA [169]. An innovative in situ thermosensitive hydrogel consisting of Persian gum (PG) blended with methylcellulose (MC) was synthesized and further incorporated with taxifolin (TAX)-loaded halloysite nanotubes (HNTs). The results showed that MC mixed with 1% PG and 3% HNTs possessed excellent mechanical strength and osteoconductive behavior using MG-63 cells [170].

The regeneration of a segmental bone defect was analyzed using composite scaffolds comprising chitosan, chondroitin sulfate, and gelatin blended with nano-bioglass at varying concentrations (4% *w*/*v*, 8% *w*/*v*, and 12% *w*/*v*) that were fabricated by polyelectrolyte complexation/phase separation followed by resuspension in gelatin. In vitro results showed that the developed scaffolds were biocompatible, with better bone regeneration in animal models [171]. Three-dimensional porous scaffolds were developed by the thermally induced phase separation method using layered double hydroxide (LDH) mixed with PCL nanocomposites reinforced with 0.1–10 wt.% LDH. The mechanical strength was improved due to LDH in the PCL scaffold. It possessed an interconnected porous structure measuring 5–150 µm, with an increase in the mineral deposition, supporting the viability, adhesion, and proliferation of human bone-marrow-derived mesenchymal stem cell (hBMSC)-seeded scaffolds [172].

### 8.2. Electrohydrodynamic Methods

**Figure 11:**
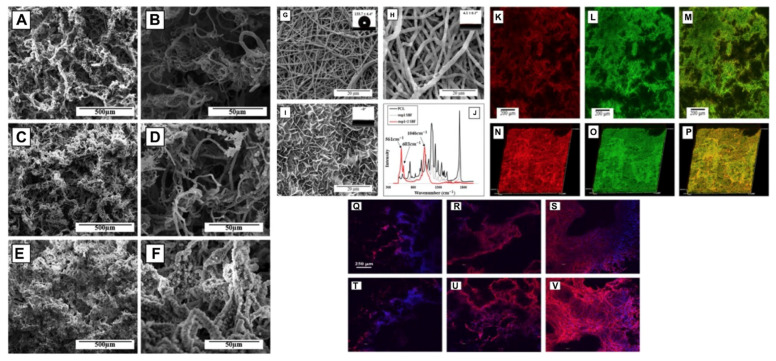
Composites comprising PCL developed by an electrospinning-based thermally induced self-agglomeration (TISA) technique. SEM morphology displaying representative PCL-3D scaffolds in low (**A**–**C**) and high (**D**–**F**) magnification. Rows top to bottom visualize neat PCL, step 1 coating, and step 2 SBF coating. FE-SEM image and contact angles of NF PCL mats before immersing in either SBF step (**G**), after step 1 (**H**), and after both steps (**I**). ATR spectra of NF PCL mats prior to and after SBF treatment (**J**). Confocal microscopy of PCL/HA scaffolds of individual layers (**K**–**M**) and 30 μm 3D-cross sections (**N**–**P**). Fluorescent dyes were introduced into step 1 SBF with rhodamine B in red colour and FITC-BSA in green. C2C12 morphologies on TISA (top row) and TISA/HA composite (bottom row) scaffolds, after 24 h (**Q**,**T**) and 72 h (**R**,**U**) of culture. (**S**) and (**V**) show a 100 μm z-stack of cell morphologies after 72 h of culture in individual scaffold. Reprinted with permission from Ref. [174], Copyright 2021, Elsevier Ltd.

In 2019, C. Buga et al. prepared a powder suspension of CaSi (calcium silicate) using TEOS (tetraethyl orthosilicate) and calcium nitrate as the precursors. Titanium plates were polished using the CaSi suspension by the electrospray method. The results showed that the deposition of a uniform CaSi layer on a titanium substrate improved the annealed temperature, granting it better corrosion resistance. Overall, the ESD (electrospray deposition) method is a simple method to form a uniform CaSi layer on a Ti substrate [173]. A biomimetic 3D nanofibrous (NF) scaffold made of PCL and HA using an electrospinning-based thermally induced self-agglomeration (TISA) technique was produced (Figure 11). The PCL/HA-TIA scaffolds were capable of encapsulating drugs. In vitro results using C2C12 cells showed superior osteogenic differentiation and lower burst release by the combined effect of phenamil and BMP2 in comparison to physically surface-adsorbed phenamil [174].

A blend polymer solution of 8% PVA, 7% aqueous solution of carboxymethyl chitosan (CMCh) at different proportions, and 2 wt% graphene oxide (GO) was used as a sheath, whereas the core material consisted of 30 wt% 4-arm PCL with 2% (*w*/*w*) Zn-Curcumin complex (Zn-CUR). The results showed that the electrospun scaffolds incorporated with Zn-CUR were biocompatible in MG-63 osteoblastic cells. Moreover, there was improved cell adhesion and proliferation, with positive antibacterial activity and calcium and mineral production [175].

In 2021, Zixu Wang et al. fabricated gelatin nanofibers by solution electrospinning, followed by the deposition of a PCL melt electrospinning writing (MEW) layer. The results showed that the developed composite scaffold possessed better mechanical properties and a favorable ECM-mimicking 3D microenvironment compared to the conventional scaffold. In vitro results using Saos-2 cells showed that the scaffold supported cell adhesion and proliferation, with better osteogenesis [176]. In 2021, Ece Guler et al. analyzed the effect of vitamin D_3_, vitamin K_2_, and magnesium at varying concentrations on PLA, TCP (tricalcium phosphate), and PCL electrospun fibers on the osteoinductive behavior, resulting in better osteogenic differentiation on mesenchymal stem cells, with improved expression of Runx2, BMP2, and osteopontin and suppression of PPAR-γ and Sox9 [177].

The PLGA shell layer was modified using fish collagen (FC), and the PCL core layer was loaded with baicalin (BA) to obtain a PLGA shell layer (PFC)/PCL-BA fibrous scaffold. The continuous release of BA showed osteogenic differentiation on bone mesenchymal stem cells, with the regulation of the macrophage phenotype transition. Favorable angiogenesis with accelerated bone formation in a rat model showed the developed core–shell-structured nanofiber is suitable for vascularized bone regeneration [178].

Exosomes, as an osteoinductive factor, support bone regeneration. Exosomes from human adipose-derived stem cells (ADSCs) were isolated. The electrospun scaffolds were fabricated via coating isolated exosomes on a synthesized magnesium–gallic acid MOF (metal–organic framework). The coated scaffolds stabilized the bone graft environment, with superior osteogenic differentiation and bone formation in an in-vivo experiment [179].

### 8.3. Additive Manufacturing Method

**Figure 12:**
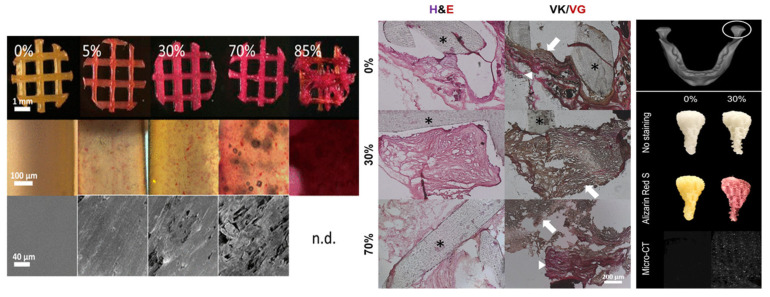
3D-printed hybrid scaffolds. Top view depicting positively stained scaffolds for Alizarin Red S in all cases except pure polycaprolactone case. Middle image showing magnified images of stained scaffold struts describing the punctate stain of the mineralized particles within the PCL. Bottom view showing SEM of strut surfaces displaying rougher surface topographies in the more concentrated hybrid scaffolds. Histological studies of excised constructs. Cellularity under H & E stain (left) as well as bone (black/dark brown) and osteoid (red) formation under the von Kossa and van Gieson stains (right) is obvious. Asterisks represent scaffold struts. In the von Kossa and van Gieson stains, note the presence of both osteoid (red, arrowheads) and mineralized tissue (red/brown, arrows), signifying active mineralization appearing within the constructs. Anatomical shape printing of pure and hybrid scaffolds. Middle image showing human temporomandibular joint condyle was obtained and printed into anatomically shaped, porous scaffolds. Scaffolds were subject to ARS staining to confirm and visualize the presence of mineralized particles in the hybrid scaffold. Bottom view represents the MicroCT scans to check the presence of mineralized particles in the 30% DCB:PCL scaffolds. There were no mineral particles present in pure PCL scaffold. Reprinted with permission from Ref. [180], Copyright 2019, Elsevier Ltd.

ECM-based 3D-printed hybrid scaffolds were created for the first time using a decellularized bone (DCB) matrix blended with polycaprolactone (Figure 12). The DCB/PCL scaffolds possessed osteoinductive properties when seeded with human ADSCs. They also showed enhanced calcification due to soluble phosphate. An in vivo study on a critically sized murine calvarial defect model supported better bone regeneration than PCL alone 1 and 3 months after implantation [180].

In 2018, for the first time, Shuai C. et al. aimed to control the pore structure, mimicking the bone microenvironment, and fabricated GO/PLLA scaffolds by combining additive manufacturing and the chemical etching process [181]. The porous PLA/PCL/HA composite scaffolds were fabricated by an indirect 3D printing technique with freeze drying and showed a favorable pore size of 160 µm and 1.35 MPa Young modulus, with increased cell viability and mineral deposition [182,183]. Another study reported the development of zinc porous bone scaffolds by combining additive-manufacturing-produced templates and casting. The obtained porous Zn scaffolds revealed interconnected porous structures with outstanding antibacterial properties and biocompatibility [184].

**Figure 13:**
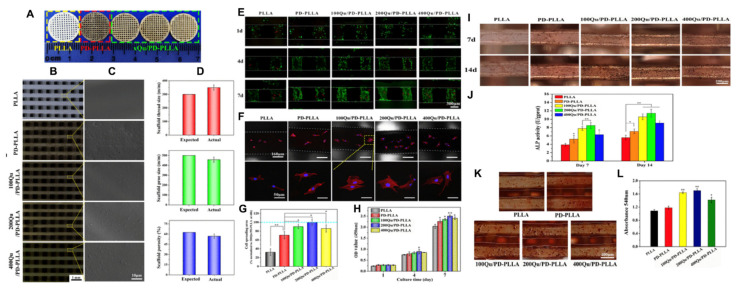
Three-dimensional printed scaffolds of PLLA, PD-PLLA, and Qu/PD-PLLA with their stereomicroscope images. (**A**) Camera images, (**B**) stereomicroscope images, and (**C**) SEM images of the 3D-printed scaffolds of PLLA, PD-PLLA, 100Qu/PD-PLLA, 200Qu/PD-PLLA, and 400Qu/PD-PLLA. (**D**) Printing reproducibility and accuracy were studied by quantification of thread diameter, pore size, and porosity determined by image analysis from stereomicroscope pictures. (**E**) Live/dead fluorescent staining images of MC3T3-E1 cells on the PLLA, PD-PLLA, and Qu/PD-PLLA scaffolds after 1, 4, and 7 days of culture. The confocal laser scanning microscopy images of (**F**) morphology and (**G**) quantification of spreading area of the MC3T3-E1 cells after culturing on the PLLA, PD-PLLA, and Qu/PD-PLLA scaffolds for 48 h. The OD value of the MC3T3-E1 cells culturing on the PLLA, PD-PLLA, and Qu/PD-PLLA scaffolds for (**H**) 1, 4, and 7 days. (**I**) ALP staining, and (**J**) ALP activity of MC3T3-E1 cells after culturing on the PLLA, PD-PLLA, and Qu/PD-PLLA scaffolds for 7 and 14 days. (**K**) Alizarin red staining and (**L**) quantitative result of MC3T3-E1 cells after culturing on the PLLA, PD-PLLA, and Qu/PD-PLLA scaffolds for 21days. Reprinted with permission from Ref. [185], Copyright 2019, Taylor & Francis.

The researchers used quercetin (Qu)-loaded 3D-printed PLLA scaffolds modified by a PDA (polydopamine) adhesive coating for bone tissue engineering application (Figure 13). The results revealed improved hydrophilicity and compressive properties due to the immobilization of PDA and Qu on the PLLA scaffolds. In vitro results using MC3T3-E1 cells showed better cell attachment and proliferation and the upregulation of alkaline phosphatase (ALP) activity, calcium nodules, osteogenesis-related genes, and protein expression as well as the controlled release of quercetin [185].

### 8.4. Others

The researchers fabricated a microsphere-based scaffold system by preparing microspheres using an alginate gel solution, a GO solution, and a dexamethasone solution under constant stirring, followed by cross-linking. The results showed that Alg-GO-Dex (alginate–graphene oxide–dexamethasone) microspheres depicted good porosity and in vitro biomineralization, sustained drug release, and improved biocompatibility on MG-63 cells compared to control. More mineralization was observed in Alg-GO-Dex microspheres than in Alg-GO microspheres [16].

**Figure 14:**
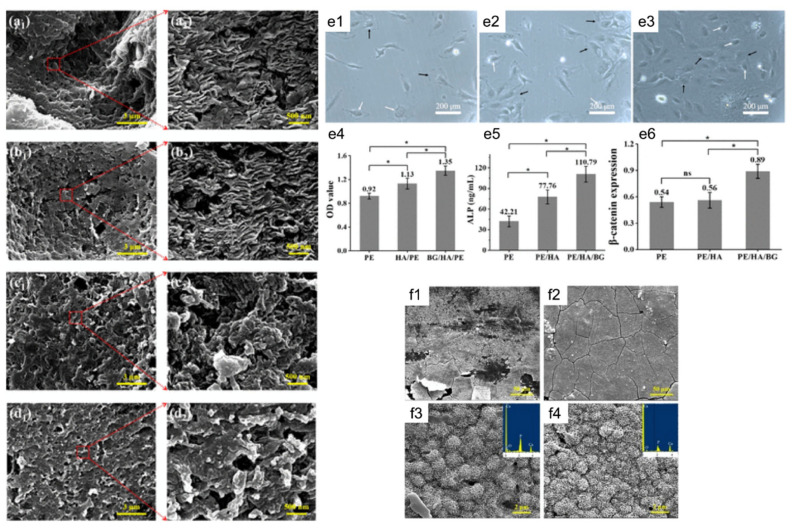
Bone substitute formed by structuring injection molding. FE-SEM images showing crystalline morphology of (**a1**,**a2**) structured HA/PE, (**b1**,**b2**) structured BG/HA/PE, (**c1**,**c2**) normal HA/PE, and (**d1**,**d2**) normal BG/HA/PE. MC3T3-E1cells adhesion and spreading on (**e1**) structured PE, (**e2**) structured HA/PE, and (**e3**) structured BG/HA/PE after culturing for 7 days. (**e4**) CCK-8 results, (**e5**) ALP results and (**e6**) Western blot analysis. The intensities of each test protein bands in (f) were normalized. SEM images of the surface of (**f1**,**f3**) structured HA/PE and (**f2**, **f4**) structured BG/HA/PE after immersion in SBF for 21 days: magnification of (**f1**,**f2**) 1000× and (**f3**,**f4**) 10,000×. The insets of c and d are the EDX spectrum of the sample surface. Reprinted with permission from Ref. [186], Copyright 2020, SAGE.

The structuring injection molding method was used to fabricate polyethylene (PE) composites mixed with BG/HA as bioactive fillers (Figure 14). The results showed an interlocked shish kebab pattern, similar to well-aligned collagen fibers in natural bone. The mechanical strength and toughness of the BG/HA/PE composites were better than in those fabricated using BG alone. An in vitro study using MC3T3-E1 osteoblast cells revealed superior cell adhesion and proliferation. The formation of apatite on the scaffold surface was dense compared to the HA/PE composite [186].

The bone scaffolds fabricated by several techniques and polymers and their structures, cells used, advantages, and disadvantages are summarized in Table 1.

## 9. Conclusions and Future Perspective

BTE aims to support bone regeneration by the fabrication of an ECM-mimicking bone scaffold, providing a complex microenvironment/nanoenvironment that provides cell adhesion and migration. There are many strategies to treat bone defects, which is challenging both for tissue engineers and orthopedic surgeons. Additionally, the choice of polymers used for scaffold fabrication is a key objective to attain biomimetic platforms. To attain this, various fabrication techniques are being developed to obtain a native bone tissue that should be porous, of controlled pore size, biocompatible, biodegradable, osteoconductive/osteoinductive, and possess the mechanical strength to persist at the implantation site of the patient. Recently, biofabrication approaches such as fiber-forming electrospinning techniques as well as 3D scaffold-forming additive manufacturing techniques have attracted researchers’ attention in the tissue engineering field. The production of electrospun fibers provides a diverse internal microstructure to the scaffold, but the limited thickness of mats and the maintenance of a constant fiber diameter is a critical task. Three-dimensional printing offers precise control over bone microarchitecture. However, the in vivo applications of integrated growth factors, bioactive materials, biomimetic scaffold designs, and functionalization techniques on the complex scaffold should be analyzed in future studies. Moreover, this printed structure should self-transform into a prescribed shape and function according to the stimulus of the bone microenvironment. To attain this, the 4D printing method fabricates shape-changing smart scaffolds that may provide conciseness in the in vivo application upon implantation. The idea behind scaffold fabrication is not only to mimic the natural bone tissue but also to encourage the maturation of bone tissue. In conclusion, emphasis should be placed on the incorporation of immunomodulatory and angiogenesis properties for the improvement in in vivo applications with a better understanding of the local biomechanical environment.

## Figures and Tables

**Table 1:** Lists of fabricated scaffolds used in bone tissue engineering, polymers, and their structures, cells used, advantages, and limitations. (**A**) Solvent casting. (**B**) Freeze drying. (**C**) Gas foaming. (**D**) Phase separation method. (**E**) Hydrogels. (**F**) Microspheres. (**G**) Electrospinning. (**H**) Additive manufacturing techniques.

(A)						
Scaffolds	Polymers	Structures	Cells Used	Advantages	Limitations	References
Chitosan(CS)/silk fibroin(SF)/reduced graphene oxide (rGO) composite membranes	CS SF rGO	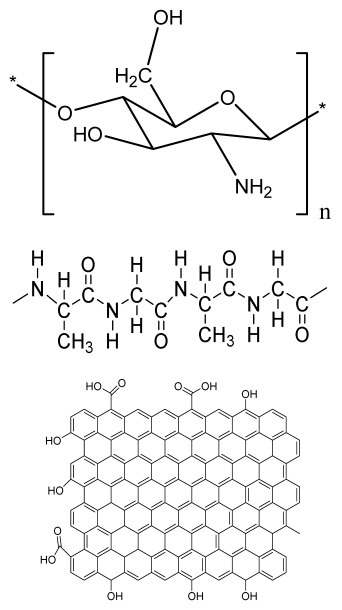	G-292 cells	Decrease in hydrophilicity, swelling, and degradability with an increase in SF content, increase in tensile strength and hydrophilicity due to increase in rGO concentration	No in vivo study Osteoconductivity study of scaffolds should be analyzed. In vitro study should be analyzed using other bone cell lines such as MG-63 and MC3T3-E1 cells.	[187]
PDA-modified BMP2- immobilized PLGA/MH composite scaffold	PLGA Magnesium hydroxide (MH) PDA	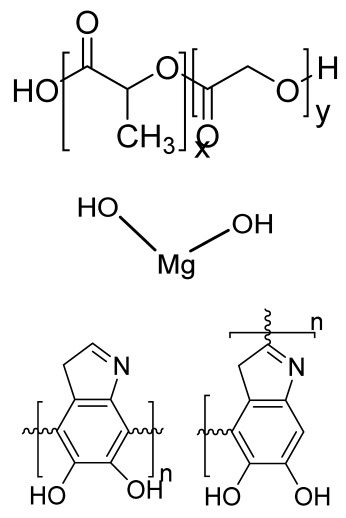	MC3T3-E1 cells	Better hydrophilicity, neutralization effects, and degradation performance and enhanced BMP2 loading efficiency supported proliferation and osteogenic differentiation and BMP2-induced bone formation.	Short-term in vivo study (4 weeks) In vitro study for a short duration (7 days) using PLGA (synthetic polymer)	[188]
Copper-hydroxyapatite/ chitosan/polyvinyl pyrrolidone composite	Chitosan Polyvinyl pyrrolidone Copper-hydroxyapatite	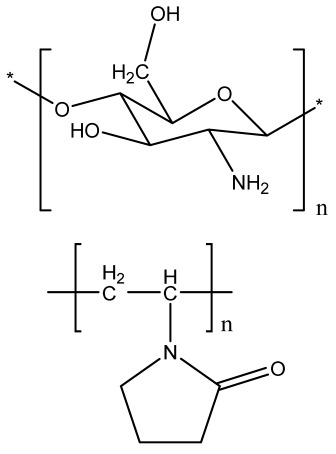	Human osteosarcoma cell line	High porosity and tensile strength, lower swelling percentage, possesses antimicrobial activity and hemocompatibility, helps in the formation of apatite, good biocompatibility and cell attachment	No in vivo study The effects of osteogenic markers using the developed scaffold should be studied. In vitro biodegradation study using the scaffolds is required.	[189]
PCL–porcine bone powder (BP) composites reinforced with PLA-CS microfibers	BP PLA PCL CS	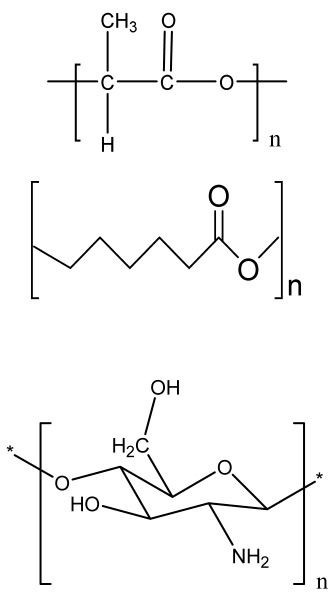	-	Suitable mechanical properties and effective bactericidal efficiency	Possibility to fabricate electrospun composites using PCL-BP reinforced with CS-PLA microfibers In vitro studies required Needs animal sacrifice Ethical issues in some countries	[190]
Gelatin (G)–HA scaffolds containing vitamin D (VD)-loaded graphene oxide	G HA GO	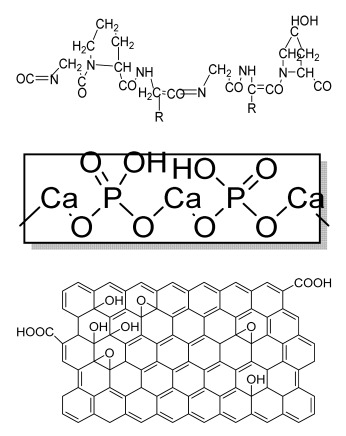	Buccal fat pad derived stem cells (BFPSCs)	Better encapsulation efficiency and mechanical properties, porosity percentage and density comparable to spongy bone, good cell adhesion and cell viability, possesses ALP activity	Developed scaffolds should be examined on animal models. Toxicity of graphene oxide at high concentration (2%)	[191]
**(B)**						
**Scaffolds**	**Polymers**	**Structures**	**Cells Used**	**Advantages**	**Limitations**	**References**
Alginate and Mg-doped calcium phosphate fillers	Sodium alginate HAP Magnesium Nitrate Hexahydrate	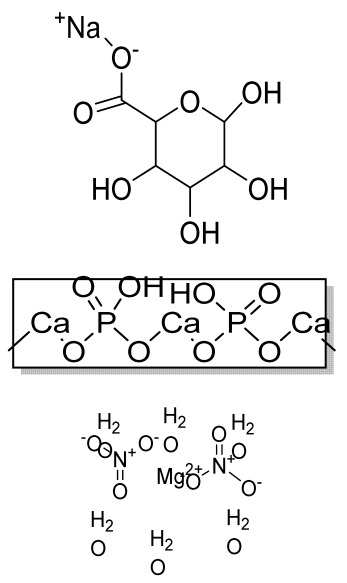	-	Highly porous and open connected pores; Better mechanical property due to Ca^2+^ ions compared to the previous scaffolds	In vitro and in vivo study needed Swelling and degradation behavior required	[192]
Monetite-nanoparticle-impregnated gelatin-based composite scaffold	Gelatin Monetite	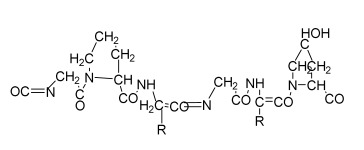	MG-63	Increase in compressive strength and better bioactivity compared to gelatin scaffolds; higher biomineralization ability, exhibits osteoinduction	Long processing time The developed scaffolds should be analyzed in animal models. Osteogenic differentiation using scaffold should be analyzed.	[193]
Gellan–chitosan scaffolds modified with calcium silicate	Calcium silicate Gellan CS	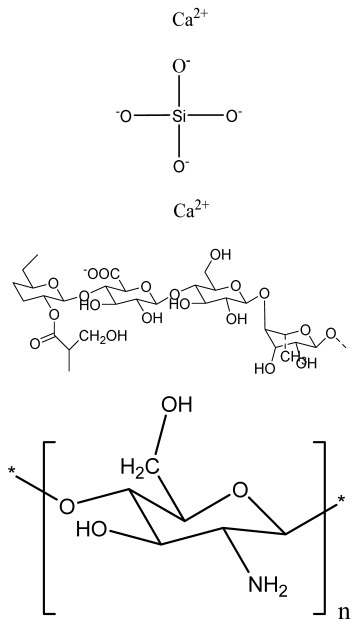	HBMSCs cells	Good cell attachment, increased proliferation and viability, supported bone mineralization, showed osteoinduction potential	In vivo experiments required for further human applications Mechanical strength using the scaffold is needed.	[194]
Collagen (Col)–rGO scaffolds	Col rGO	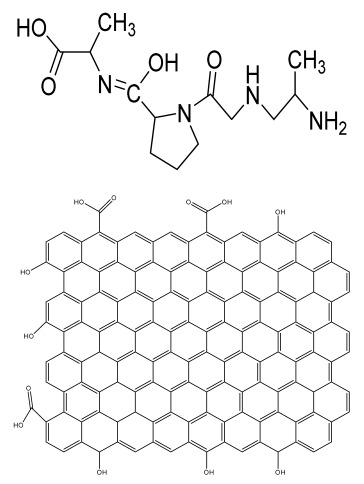	hBMSCs cells	Increased mechanical strength due to rGO; nontoxic, with better viability and proliferation of cells; increased bone formation in mouse models within 12 weeks of implantation	In vivo study should be performed on higher animal models. The effects of developed scaffolds should be analyzed using growth factors.	[195]
Gelatin/chitooligosaccharide/ demineralized bone matrix composite scaffold	G Chitooligosaccharide (COS) bone matrix (BM)	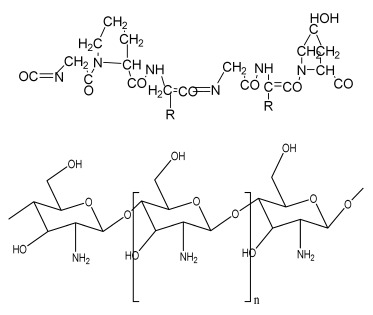	Mesenchymal stem cells	Improved cell attachment with proliferation on scaffold, mineralization until 8 weeks, supported in vivo ectopic bone formation	Osteoconductive and osteoinductive features required for the confirmation of increased bone formation.	[196]
** (C) **						
**Scaffolds**	**Polymers**	**Structures**	**Cells Used**	**Advantages**	**Limitations**	**References**
PCL-based PU foam scaffolds	PCL triol PU Gelatin	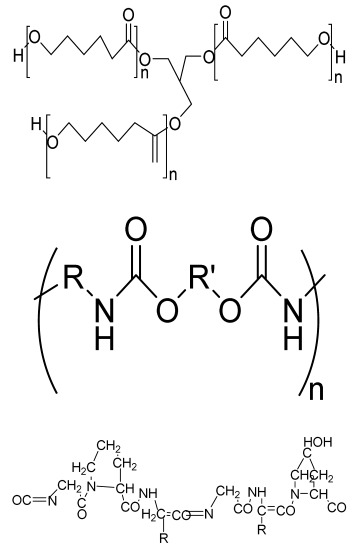	MC3T3-E1 cells	Highly porous structure, high compression strength, ductile and flexible, low toxicity, high ALP activity	The application of optimized PU scaffold for specific tissue is needed in an animal model for future analysis	[197]
Ca-3D@PCL-CL24 (3D multilayered polycaprolactone/cellulose (CL) scaffold)	PCL CL Calcium hydroxide particles	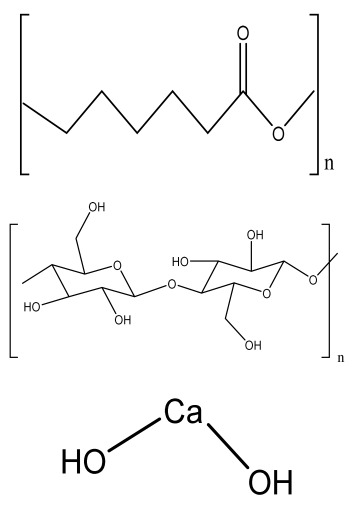	MC3T3-E1 cells	Better mechanical and thermal properties compared to the control, enhanced cell growth and mineralization	The function of the scaffold in animal models should be analyzed in a future study. Long degradation time using synthetic polymer-based scaffold	[198]
HA/PU composite porous 3D scaffold	HA PU	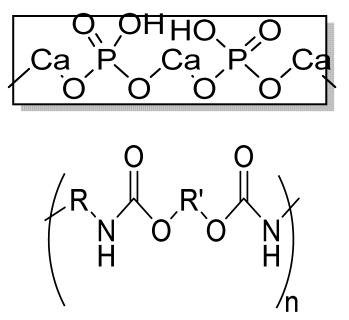	-	Promotes bioactivity, superior mechanical properties, satisfactory degradation time (12 weeks)	In vitro studies are required on bone cells. Osteoinductive behavior should be analyzed.	[199]
**(D)**						
**Scaffolds**	**Polymers**	**Structures**	**Cells Used**	**Advantages**	**Limitations**	**References**
Deferoxamine (DFO)-loaded poly (glycerol-co-sebacic acid-co-L-lactic acid-co-polyethylene glycol) (PGSLP)-based composite scaffolds	Poly (glycerol-co-sebacic acid-co-L-lactic acid-co-polyethylene glycol deferoxamine Gelatin	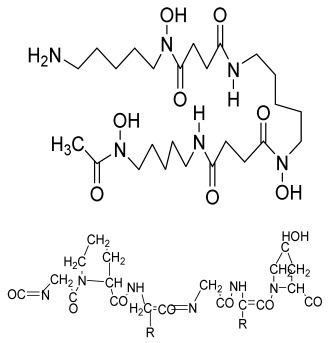	Human umbilical vein endothelial cells (HUVECs)	Supported vascular formation, enhanced bone regeneration, supported cell adhesion and migration, promoted osteogenesis and angiogenesis, enhanced mineral nodule formation and vascular formation and promoted bone formation in rat model	Optimization of the scaffold is needed in a higher animal model in a future study.	[200]
HA/PLA/ASA/GO (hydroxyapatite/polylactic acid/aspiringraphene oxide/) drug-loaded biomimetic composite scaffold	PLA HA GO aspirin	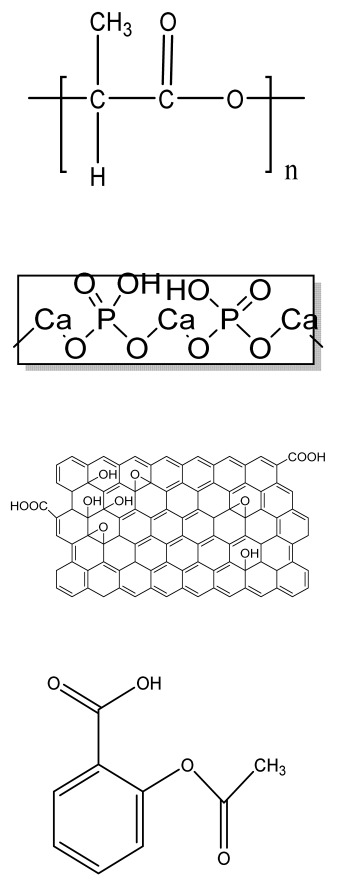	MC3T3-E1	Hydrophilic scaffold with good bioactivity, hemocompatibility, cytocompatibility, and sustained drug release	The inhibitory effect of ASA on bone cells may hinder bone regeneration.	[201]
**(E)**						
**Scaffolds**	**Polymers**	**Structures**	**Cells Used**	**Advantages**	**Limitations**	**References**
Transforming growth factor-β3-loaded Sil-MA (methacrylated silk fibroin) hydrogel	SF	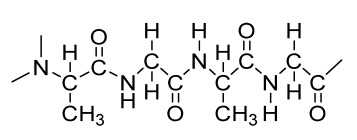	BMSCs	Osteochondral regeneration, better lateral integration, good adhesive property, marginal sealing effect, promotes chondrocyte migration and differentiation	In vivo analysis should be performed on higher animal models with long duration. Hydrophilicity and degradation study required	[202]
Gelatin methacrylate (GelMA)/Bone meal powder (BP) composite hydrogels	GelMA BP	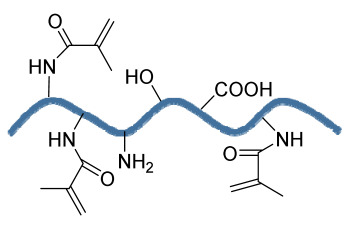	MC3T3-E1	Improves osteoinductivity and mechanical performance and supports cell differentiation, biocompatibility, and biodegradation properties	The effect of the hydrogel should be analyzed in higher animal models. The developed scaffold should be analyzed for the delivery of small molecules, for example, therapeutics and growth factors.	[203]
**(F)**						
**Scaffolds**	**Polymers**	**Structures**	**Cells Used**	**Advantages**	**Limitations**	**References**
Mg-Ca silicate microspheres encapsulated in PLGA	Sodium alginate PLGA	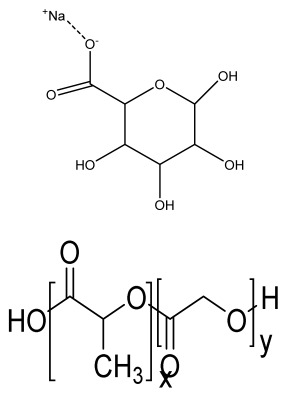	Dental pulp stem cells	Limited the burst release of the vancomycin and drug concentration was above the critical value inhibiting *S.aureus* growth. PLGA-coated akermanite microspheres showed highest cell viability	The effects of developed microspheres should be analyzed in animal models and should also be optimized using other drugs.	[204]
HA-SF-PLGA hybrid porous microspheres	HA PLGA SF	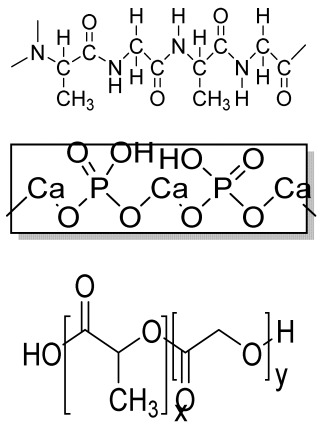	Human periodontal ligament stem cells (hPDLSCs)	Enhanced viability, proliferation, and osteogenic differentiation and better tissue repair efficacy	In vivo study required. Osteoinductive behavior should be analyzed.	[205]
**(G)**						
**Scaffolds**	**Polymers**	**Structures**	**Cells Used**	**Advantages**	**Limitations**	**References**
Bioceramic PCL scaffold containing metallic oxides	PCL Hybrid TiO_2_@ZrO_2_ composite	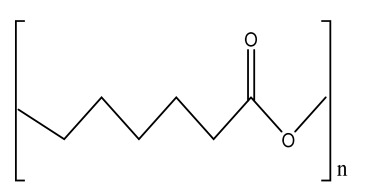	MC3T3-E1 cells	Excellent antibacterial activity, improved load-bearing ability, hydrophilicity, and biomineralization, better cell-to-cell interactions, enhanced proliferation and regeneration, and good biocompatibility with osteoinductive abilities	No in vivo study Long-term degradation of PCL Short-term in vitro study (5 days), as PCL was used, which is a synthetic polymer	[206]
Poly (3-hydroxybutyrate(PHB)/starch electrospun scaffold	PHB Starch	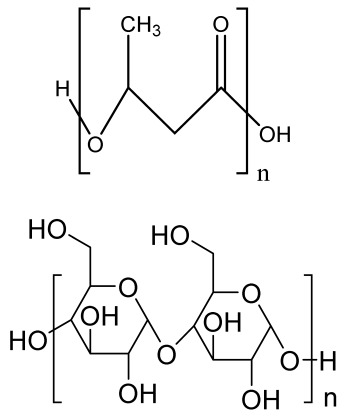	MG-63 cells	Improved tensile strength, degradation, and hydrophilicity due to starch, better viability and proliferation of the electrospun scaffolds than PHB scaffold	The effect of growth-factor-incorporated electrospun scaffold should be analyzed in future study.	[207]
Chitosan nanofibrous scaffolds modified by polydopamine (NFs-PDA)	Chitosan Dopamine hydrochloride	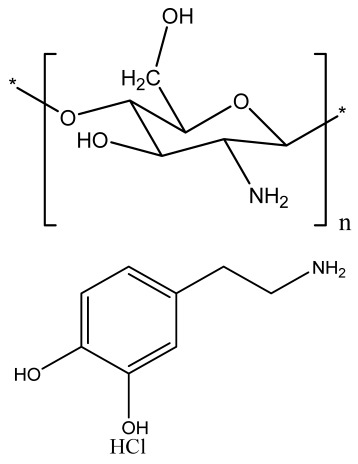	MT3C3-E1 cells	Supported structural stability of fabricated nanofibers in PBS and improved cytocompatibility and in vitro biomineralization	Investigation of the in vivo process for biological action with replacement of NF with newly formed bone	[208]
Porous magnetic PCL/Fe_3_O_4_/icariin (ICA) 3D scaffold	Fe_3_O_4_ MNPs ICA PCL fibers	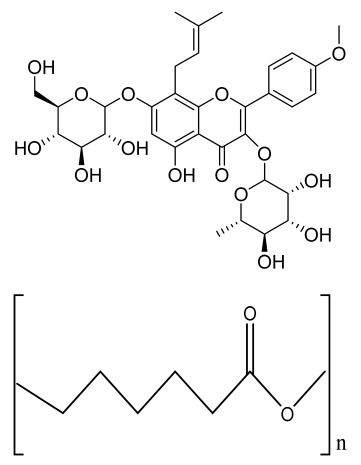	MC3T3-E1	Improved cell proliferation viability compared to 2D membrane, increased cell viability, and superior cell infiltration, internal collagen deposition, and angiogenesis	The developed scaffold should be analyzed in higher animal models. Mechanical strength and degradation behavior required of developed scaffold	[209]
PGS-PHB scaffold	Poly (glycerol sebacate) PHB	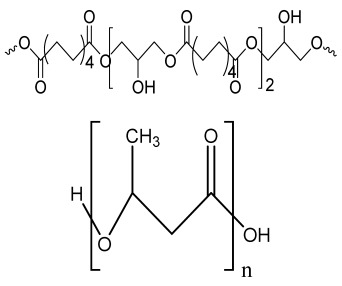	ADSCs	Good porosity, higher protein adsorption capacity than control, biocompatible, better ALP activity, calcium production, and expression level of bone-related genes	The effect of functionalized PGS-PHB scaffold should be analyzed in future study.	[210]
**(H)**						
**Scaffolds**	**Polymers**	**Structures**	**Cells Used**	**Advantages**	**Limitations**	**References**
miRNA-activated hydrogel scaffolds (MAHSs)	Gelatin Alginate	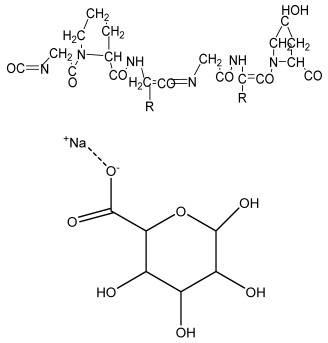	hMSCs (human bone mesenchymal stem cells)	Sustained release of miR-29b, accelerated bone regeneration, induced osteogenesis and new bone formation	Short term in vivo study (4 weeks) Use of glutaraldehyde as a cross-linker may be toxic to cells.	[211]
PCL/nHA scaffolds	PCL nHA Polyglycolic acid	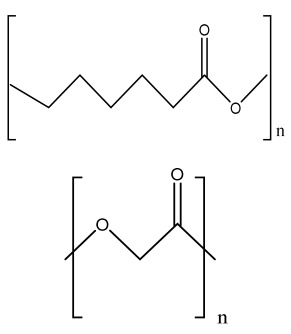	Mouse fibroblast cells ((L-929)	Improved tensile properties and compressive strength, increased hydrophilicity, increased adhesion and mechanical property, and nontoxic, suitable for bone tissue engineering	In vivo study required for the developed scaffold. Osteoinductive and osteoconductive behavior should be analyzed. Mineralization study required.	[212,213,214]
ASP (abalone shell particles)-embedded PCL scaffolds	PCL	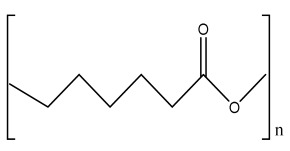	MG63 cells	Increased mechanical properties, improved absorption of cell proteins, supported cell viability and proliferation, high ALP activity, supported bone regeneration	The developed method is costly. In vitro biodegradation study required.	[213,215,216]
Poly (vinyl alcohol)/ polylactic acid/hydroxyapatite composite scaffolds	PVA PLA HA	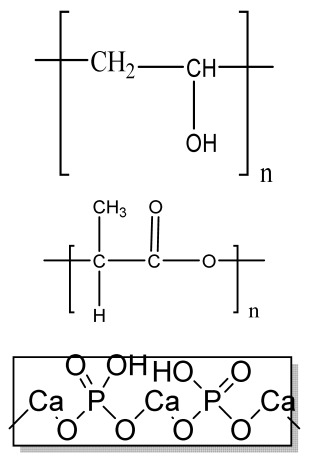	L929 cells	Increased compressive strength and compressive modulus, good bone formation and mineralization ability, excellent biocompatibility	In vitro study performed for very short duration (72 h). In vivo study should be performed in future study.	[214,217,218]
Biomimetic 3D cell-laden construct	Collagen β-TCP	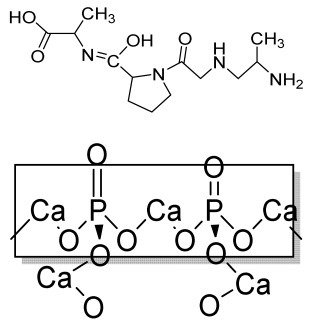	hASCs, HUVECs	Positive angiogenic phenotype; improved new bone formation and angiogenesis.	The effect of other cell lines on 3D construct should be analyzed. The biomimetic scaffolds should be analyzed for clinical trials in higher animal models.	[219,220,221]
Magnesium-based nanocomposite bioink material	Magnesium hydroxide nanoparticles (Mg) PCL	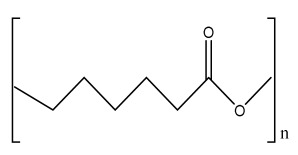	Human mesenchymal stromal cells (hMSCs)	Enhanced osteogenic differentiation and bone-specific matrix deposition, accelerates degradation rate of scaffold compared to PCL, and supported bone ECM deposition	The developed scaffolds should be analyzed in animal models in future study.	[216,222]
